# Data‐Driven Theoretical Modeling of Centrifugal Step Emulsification and Its Application in Comprehensive Multiscale Analysis

**DOI:** 10.1002/advs.202411459

**Published:** 2025-02-08

**Authors:** Xin Wang, Xiaolu Cai, Chao Wan, Huijuan Yuan, Shunji Li, Yiwei Zhang, Ran Zhao, Yuxi Qin, Yiwei Li, Bi‐Feng Liu, Peng Chen

**Affiliations:** ^1^ The Key Laboratory for Biomedical Photonics of MOE at Wuhan National Laboratory for Optoelectronics‐Hubei Bioinformatics & Molecular Imaging Key Laboratory Systems Biology Theme Department of Biomedical Engineering College of Life Science and Technology Huazhong University of Science and Technology Wuhan 430074 China

**Keywords:** centrifugal step emulsification, CFD, digital assay, droplet microfluidics, POCT

## Abstract

Tailored droplet generation is crucial for droplet microfluidics that involve samples of varying sizes. However, the absence of precise predictive models forces droplet platforms to rely on empiricism derived from extensive experiments, underscoring the need for comprehensive modeling analysis. To address this, a novel customized assembled centrifugal step emulsifier (CASE) is presented by incorporating a “jigsaw puzzles” design to efficiently acquire large‐scale experimental data. Numerical simulations are utilized to analyze fluid configurations during step emulsification, identifying a key connection tube that determines droplet size. By training and verifying with the experimental and simulation datasets, a comprehensive theoretical model is established that allows for the preliminary design of the droplet size and generation frequency with an average error rate of 4.8%, successfully filling a critical gap in existing field. This predictive model empowers the CASE to achieve all‐in‐one functionality, including droplet pre‐design, generation, manipulation, and on‐site detection. As a proof of concept, multiscale sample analysis ranging from nanoscale nucleic acids to microscale bacteria and 3D cell spheroids is realized in the CASE. In summary, this platform offers valuable guidance for customized droplet generation by centrifugal step emulsifiers and promotes the adoption of droplet microfluidics in biochemical assays.

## Graphical abstract

A customized assembled centrifugal step emulsifier is utilized to generate an end‐point experimental dataset for droplet generation. By integrating real‐time numerical simulations, a predictive model for droplet generation is developed, meeting the diverse droplet size requirements necessary for bioassays involving molecules, bacteria, and cells.

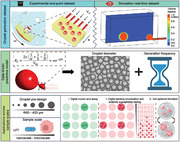

## 1 Introduction

Droplets serve as miniaturized reaction vessels with enhanced efficiency and reduced inhibition, making them widely applicable in biochemical applications, such as digital assay,^[^ ^1^ ^]^ ‐Omics analysis,^[^ ^2^, ^3^ ^]^ digital microfluidics,^[^ ^4^ ^]^ and cell culture.^[^ ^5^, ^6^ ^]^ For rapid and high‐throughput droplet generation, numerous methods have been proposed, such as T‐junction, flow focusing and co‐flow,^[^ ^7^, ^8^ ^]^ along with various droplet manipulation strategies to address different application issues.^[^ ^9^, ^10^ ^]^ However, their reliance on complex external equipment and trained personnel hinders their large‐scale adoption, especially in detection conditions where resources and staff are limited. Step emulsification can produce monodisperse droplets by leveraging the regular breakup of a continuous flow within a step configuration.^[^ ^11^, ^12^ ^]^ This methodology relies more on geometric construction and has less requirement for instrumentation and expert handling. Additionally, the integration of multiple parallel microchannels in a step emulsifier enables higher throughput droplet generation compared to other methodologies. The centrifuge is one of the most stable and commonly used instruments in laboratories and testing institutions, widely employed as a driver in droplet microfluidics.^[^ ^13^, ^14^, ^15^ ^]^ The combination of step emulsifiers and centrifugal drivers demonstrates remarkable performance. It not only mitigates the issue of droplets clustering around the nozzles, thereby preventing droplet fusion, but also enhances the applicability of step emulsification across various scenarios, particularly in point‐of‐care detection.^[^ ^16^, ^17^ ^]^

Droplet microfluidics encapsulate samples across various volume scales in different applications, from nanoscale molecules to microscale cells, necessitating different droplet sizes for optimal reaction efficiency.^[^ ^18^ ^]^ Therefore, precise and preliminary prediction of droplet sizes is essential for droplet microfluidic platforms, calling for comprehensive modeling analysis to train predictive models.^[^ ^19^, ^20^, ^21^ ^]^ Nonetheless, current theoretical modeling of centrifugal step emulsification faces challenges due to black‐box experimentation and inaccurate real‐time numerical simulations. Specifically, observing the centrifugal step emulsification process in a rotational and microscale environment is challenging without high‐speed imaging and microscopic devices, leading to reliance on end‐point observations to determine emulsification results.^[^ ^19^, ^22^ ^]^ Even though the computational fluid dynamics (CFD) can offer full range of fluid geometry configurations, idealizations in parameter design during simulation setup and practical operational complexities can result in inaccuracies.^[^ ^23^, ^24^ ^]^ Effectively linking data from both experimental and numerical simulations, and its integration into theoretical modeling, remain issues to be addressed.

**Figure 1:**
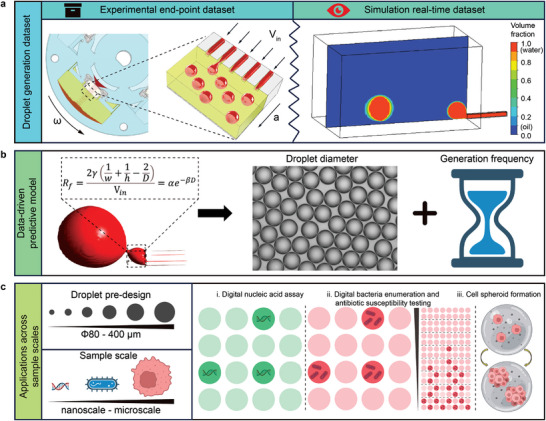
A customized assembled centrifugal step emulsifier (CASE) for droplet microfluidics. a) The flexibly adjustable characteristic of the CASE enables it to generate a large dataset from end‐point experiments. b) The CFD simulation offers real‐time observations of droplet generation and can generate another dataset. c) Combined these datasets, a data‐driven predictive model has been come up to anticipate droplet diameter and generation frequency. Accordingly, by adjusting influential parameters, droplet diameter can be pre‐designed from 80 to 400 µm, which enables the analysis cross sample scale, ranging from nucleic acids to bacteria and cancer cells.

In this study, a rapid prototyping and customized assembled centrifugal step emulsifier (CASE) fabricated by the jigsaw puzzle method was presented (**Figure** **1**a). By computer‐aided design (CAD) and assembly, relative influential parameters of the CASE can be easily adjusted as desired. As a result, a dataset from sixty different emulsification conditions have been generated experimentally. Leveraging the analysis of geometric configuration from real‐time CFD simulations, a crucial connection tube was identified as a key factor in determining droplet size, which aided in constructing a predictive model. The accuracy of the predictive model was further improved by training and verifying with the end‐point experimental dataset. Consequently, for the first time, a universal predictive model of step emulsification based on straight‐through microchannels has been developed to predict the droplet size and generation frequency across a wide range, facilitating droplet pre‐design (Figure 1b). This capability of the CASE enables analysis cross sample scale, spanning from nucleic acids to bacteria and cancer cells (Figure 1c). In conclusion, our platform enables comprehensive exploration of the potential mechanisms underlying step emulsification and holds promise for a wide array of droplet microfluidic applications.

## 2 Results

### 2.1 Assembly and Characterization

A step configuration involves a microchannel and a deeper chamber, typically fabricated using dual‐layer soft lithography,^[^ ^25^, ^26^, ^27^ ^]^ micromilling,^[^ ^28^, ^29^ ^]^ and wet etching^[^ ^30^ ^]^ technologies, which incur complex fabrication and exorbitant costs. Herein, we divided the microchannel and chamber into different “jigsaw puzzles” to construct the CASE.

**Figure 2:**
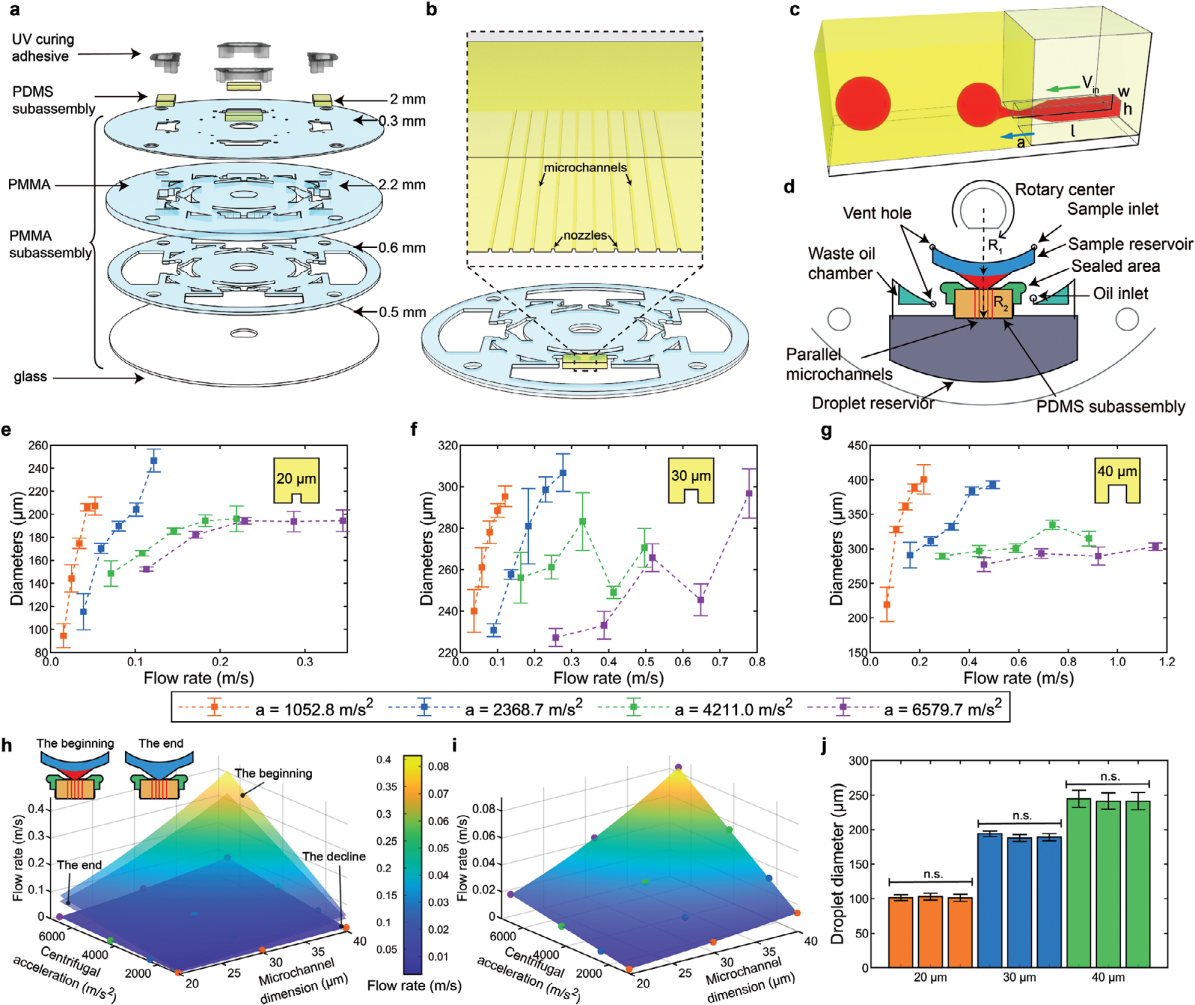
The composition of a CASE and the robustness of generated droplets. a) An exploded view of a CASE. b) Multiple parallel step constructions of a CASE. c) Schemic of droplet generation through a step construction. d) The functional chambers and channels of a CASE. e) Droplet diameters generated by 20 µm microchannels (f 30 g 40 µm microchannels) in various conditions by experimental parameter adjustment (*n* > 1000, data represented as mean ± standard deviation (SD)). h) The beginning, end and decline of *V*_in_ against different centrifugal accelerations and microchannel dimensions (the width equals to the height) when the volume of the aqueous phase was 20 µL. i) The detailed decline of *V*_in_ where the dot corresponds to conditions that in (e–g). j) The reproducibility of CASEs along different microchannels, represented as mean ± SD (*n* = 3). Statistical analysis revealed no significant differences (*p* > 0.05, indicated as n.s.).

A CASE was assembled using two types of subassemblies. First, there was a disk‐shaped polymethyl methacrylate (PMMA) subassembly with 3D millimeter‐sized chambers and channels. Second, there were rectangular polydimethylsiloxane (PDMS) subassemblies, which contain 2D, parallel, micrometer, straight‐through microchannels. The PDMS subassemblies were bonded to the bottom glass of the PMMA subassembly at reserved areas through plasma treatment. This assembly process resembles putting together a jigsaw puzzle (**Figure** **2**a). The droplet reservoirs of the PMMA subassembly were connected to the nozzles and deeper than their height, ingeniously forming many 3D parallel step configurations (Figure 2b–d). Subsequently, all the clearances connected to the air were blocked with a UV curing adhesive (Figure S1, Supporting Information). Due to the adhesive, the aqueous phase in the proximal end can only enter the droplet reservoir in the distal end through the microchannels, thereby generating droplets. The PDMS subassemblies were produced in a high‐throughput manner (40 pieces per three‐inch mold) using a cutting die (Figure S2, Supporting Information). With the assistance of CAD and rapid prototyping laser engraving technology, the rapid design and manufacturing of the PMMA subassemblies were feasible (hundreds of pieces a day). Thus, the distance between the rotational center and nozzles can be easily adjusted as needed. Additionally, three types of microchannel dimensions of the PDMS subassemblies were applied, which ensures ample flexibility in assembling a CASE. Fabricating a CASE with desired parameters using prepared subassemblies only took a few minutes, a process significantly faster than traditional fabrication methods, which can take hours or even days. Moreover, because of the common materials used, the construction cost was down to $1 per chip, with multiple CASEs in every chip.

To generate “water in oil” droplets, the aqueous and light oil phases were injected into the sample and droplet reservoirs individually through their respective inlets. An optimized oil injection procedure was employed to effectively eliminate air, preventing the influence of air bubbles during emulsification (Figure S3a, Supporting Information). The aqueous phase was driven through the microchannels by centrifugal force and met the oil phase at the nozzles, where step emulsification occurred. The microchannels were pre‐treated with hydrophobic reagents ten hours after plasma treatment to enhance emulsification performance (Figure S4, Supporting Information). However, some types of oil phase were found to cause gradual collapse of the PDMS microchannels, resulting in polydisperse droplets. Therefore, the oil phase was optimized and a mixture of n‐hexadecane and mineral oil (in a ratio of 2:3) was finally selected as the optimal oil phase (Figure S5, Supporting Information). To ensure the integrity of droplets and avoid droplet fusion during collision and stacking, 8% v/v EM180 was added to the oil phase as the surfactant.

We further investigated the homogeneity and reproducibility of the manually assembled CASE. Among all the adjustable parameters of the CASE, the microchannel dimension, the centrifugal acceleration at the nozzle (*a*) and the flow rate of the aqueous phase in the microchannels (*V*_in_) were found to ultimately dominate the droplet size. In practice, the microchannel dimension was fixed. The centrifugal acceleration remained constant under a settled rotational speed: *a*  =  ω^2^ · (*R*_1_ + *R*_2_), where ω corresponds to the angular velocity of the centrifuge, *R*_1_ corresponds to the distance between the surface of aqueous phase and the rotational center, *R*_2_ corresponds to the distance between the surface of aqueous phase and nozzles. And the *V*_in_ was related to the driving pressure, the capillary pressure, and the hydraulic resistance of the microchannels. In our design, the driving pressure was dominated by the height of the aqueous phase because the nozzles were aligned to the surface of the oil phase. Therefore, the driving pressure can be expressed as:

(1) $$\begin{array}{c} \;P_{\mathrm{aqu}}=\int_{R_{1}}^{R_{1}+R_{2}}\rho_{\mathrm{aqu}}\omega^{2}rdr=\frac{1}{2}\rho_{\mathrm{aqu}}\omega^{2}\left[{\left(R_{1}+R_{2}\right)}^{2}-{R_{1}}^{2}\right] \end{array}$$

where ρ_aqu_ corresponds to the density of the aqueous phase, *r* corresponds to the distance to the rotational center. The capillary pressure of a rectangular microchannel can be calculated as:

(2) $$\begin{array}{c} \;P_{\mathrm{cap}}=2\gamma cos\theta \left(\frac{1}{w}+\frac{1}{h}\right) \end{array}$$

where γ represents the surface tension between the aqueous and oil phase, θ represents the contact angle of the walls of a microchannel, *w* and *h* correspond to the width and height of the nozzle respectively. The hydraulic resistance of a microchannel was:

(3) $$\begin{array}{c} \;R_{\mathrm{hyd}}=\frac{12\mu l}{wh^{3}\left\{1-\frac{192h}{\pi^{5}w}\left[\sum_{i=1,3,5}^{\infty }\frac{1}{i^{5}}\tanh\left(\frac{i\pi w}{2h}\right)\right]\right\}} \end{array}$$

where μ corresponds to the viscosity of the aqueous phase, *l* corresponds to the length of the microchannel. Eventually, the flow rate of the aqueous phase in a microchannel can be calculated by combining Equations ((1), (2), (3)):

(4) $$\begin{array}{c} \;V_{\mathrm{in}}=\frac{P_{\mathrm{aqu}}+P_{\mathrm{cap}}}{R_{\mathrm{hyd}}wh} \end{array}$$

By adjusting various parameters, droplet generation under 60 different conditions was accomplished (Figure 2e–g) (Figure S6 and Table S1, Supporting Information). One data point is missing in Figure 2g due to emulsification occurring in the dripping faucet mode (refer to Section 2.2. Emulsification Procedure Analysis). Stitched images of generated droplets were acquired and analyzed programmatically (Figure S7, Supporting Information). Thanks to a multitude of parallel microchannels, ten thousand droplets can be generated in minutes with the droplet diameter ranging from 80 to 400 µm. The analysis showed excellent homogeneity in the droplet size, with a coefficient of variation (CV) as low as 0.62%. Furthermore, the results qualitatively demonstrated how the three main parameters influenced the droplet size. First, the droplet diameter increased with the microchannel dimension. Second, the diameter decreased as centrifugal acceleration increased (Figure S8, Supporting Information). Last, the droplet size grew with an increased flow rate. However, during the entire droplet generation process, where typically no‐dead volume emulsification is needed, a significant decrease in the height of the aqueous phase in the sample reservoir was observed. This indicated a decline in driving pressure, and consequently, a decline in flow rate. We revealed the decline of flow rate under these different conditions when the volume of the aqueous phase was 20 µL through detailed numerical calculations (Figure 2h) (Figure S9, Supporting Information). Comparatively, the decline in flow rate was insignificant for individual conditions, further demonstrating the excellent homogeneity (Figure 2i). Additionally, there was no difference between repeated experiments under the same conditions, indicating good reproducibility, even though the CASE was manually assembled (Figure 2j).

### 2.2 Emulsification Procedure Analysis

However, the microscale and rotational environment of droplet generation within the CASE renders the process a “black box,” making it difficult to perform quantitative analysis of the experimental results. To improve observation and analysis of fluid flow phenomena, the CFD method have been extensively employed to simulate and analyze step emulsification driven by syringe pumps.^[^ ^31^, ^32^, ^33^, ^34^ ^]^ Very few study focused on the centrifugal step emulsifiers especially for that based on straight‐through microchannels.^[^ ^19^, ^35^ ^]^ Accordingly, we devoted to figure out how a droplet is generated in the circumstance by CFD simulation.

**Figure 3:**
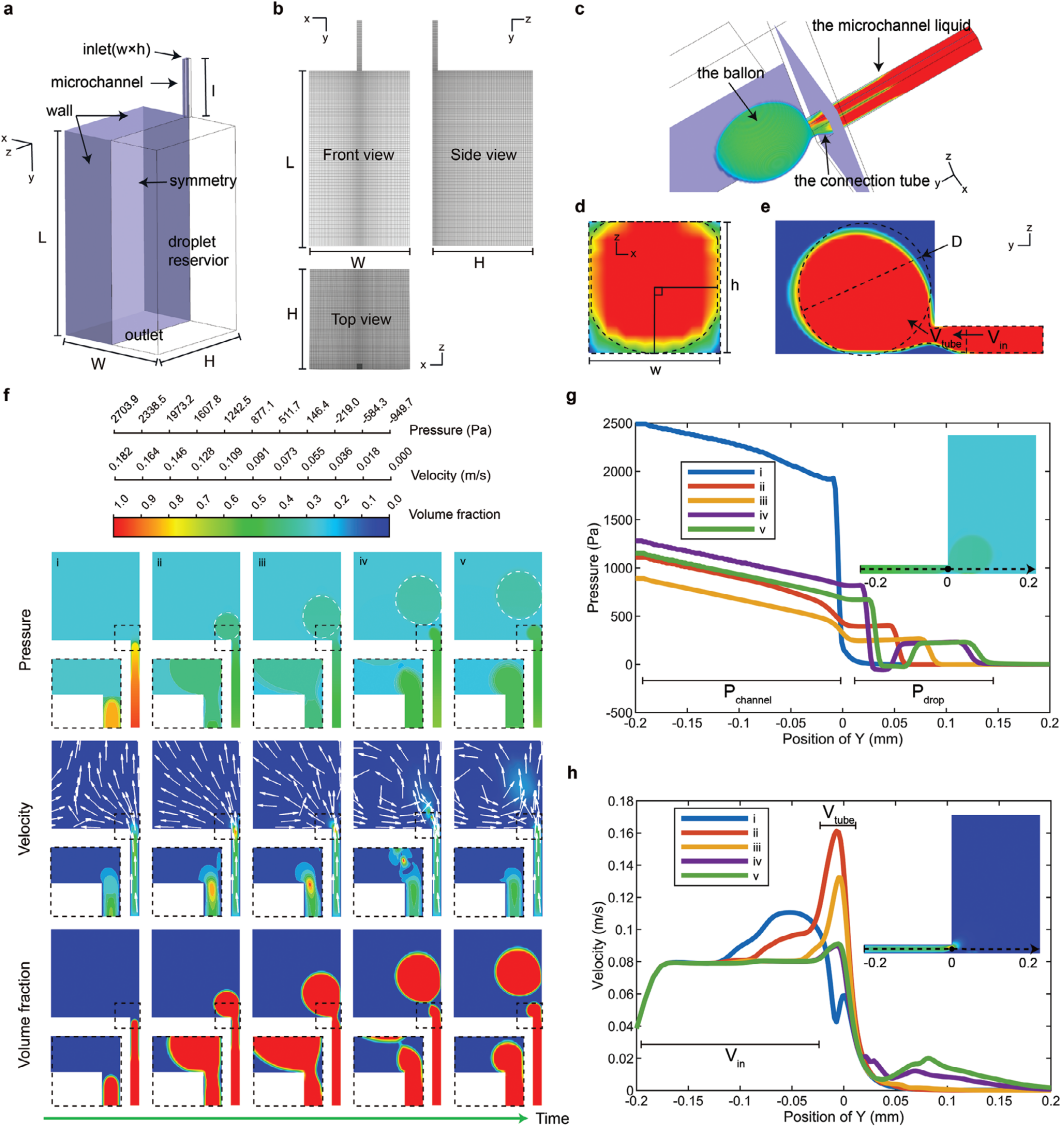
Numerical simulation of the procedure of droplet generation in the periodic dripping regime. a) The 3D model used in the simulation. b) The structured grid partition utilized in the computational domain. c) Three components of the aqueous phase near the nozzle. d) The x–z cross section of the microchannel liquid at *y* = −0.018 mm. e) The y‐z cross section of the three components at *x* = 0 mm. f) The pressure, velocity and volume fraction changes along a circle of the droplet generation: phase i. The aqueous phase flowed to the nozzle, phase ii. A ballon was growing for release of geometric constraints, phase iii. The connection tube was shrinking because of Laplace pressure difference, phase iv. A droplet was breaking up because of Rayleigh‐Plateau instability, phase v. The start of next circle. g) The pressure of the *z* = *h*/2 line in the symmetry (the dotted line in the internal illustration). Throughout an emulsification cycle, the pressure in the microchannel consistently exceeded that in the ballon, facilitating ballon expansion. The internal illustration refers to the contour of pressure at phase iii. h) The velocity of the *z* = *h*/2 line in the symmetry (the dotted line in the internal illustration). During an emulsification cycle, the flow rate in the connection tube initially increased, leading to ballon expansion, and subsequently decreased as the connection tube shrank. The internal illustration refers to the contour of velocity at phase iii.

A *w × h × l* sized microchannel was connected to a *W* × *H* × *L* sized droplet reservoir as the model (**Figure** **3**a). Half of the total domain consisted of half a million cells was utilized as the computational domain with symmetry in the *z* = 0 plane and the contact angle of walls was set to 180° to minimize the calculation burden (Figure 3b) (Figure S10, Supporting Information). The geometry and liquid parameters applied in the simulation are summarized in (Table S2, Supporting Information). As a result, when the Weber number was so low that the droplet generation was in the periodic dripping regime,^[^ ^36^ ^]^ a ballon was formed because of instantaneous relief of geometric constraints in the step (Figure 3c). To better demonstrate the droplet generation process, we divided the aqueous phase into three components: the ballon, the microchannel liquid and the connection tube. According to the Young‐Laplace equation, the difference between the oil pressure *p*_oil_ and the liquid pressure in the microchannel *p*_channel_ was constant with a fixed microchannel dimension (Figure 3d):

(5) $$\begin{array}{c} P_{\mathrm{channel}}-\;P_{\mathrm{oil}}=\gamma \left(\frac{1}{\frac{w}{2}}+\frac{1}{\frac{h}{2}}\right)=2\gamma \left(\frac{1}{w}+\frac{1}{h}\right) \end{array}$$

However, the Laplace pressure of the ballon surface was lower:

(6) $$\begin{array}{c} P_{\mathrm{drop}}-\;P_{\mathrm{oil}}=\gamma \left(\frac{1}{\frac{D}{2}}+\frac{1}{\frac{D}{2}}\right)=\frac{4\gamma }{D} \end{array}$$

where *p*_drop_ corresponds to the pressure in the ballon, *D* corresponds to the diameter of approximate sphere of the ballon (Figure 3e). The Laplace pressure difference drove the microchannel liquid flow into the ballon through the connection tube, making the ballon keep growing in size (Figure 3f,g). Theoretically, *V*_in_ was constant at the macroscale, while the liquid in the connection tube got extra motivation and flowed into the ballon with a flow rate *V*_tube_ (Figure 3h). Evidently, *V*_tube_ exceeded *V*_in_ thus the connection tube kept losing aqueous phase and shrinking. At some point, when the tube was small enough, it breaked up because of Rayleigh‐Plateau instability, producing a droplet. The procedure described above repeated over and over again in the same condition, so monodisperse droplets can be generated.

We also observed that when the Weber number was so high that the droplet generation took the form of a dripping faucet.^[^ ^36^, ^37^ ^]^ At the very beginning, a ballon was formed in a manner similar to that observed in the periodic dripping regime (Figure S11a, Supporting Information). Nevertheless, the flow rate enhancement resulting from the Laplace pressure difference was negligible compared to the swift *V*_in_. Consequently, the growth of the ballon was limited, and the conditions for Rayleigh‐Plateau instability cannot be satisified. Furthermore, a secondary bollon formed, connecting the primary ballon and the microchannel. The flow rate from the secondary ballon to the primary ballon was slightly lower than that from the microchannel to the secondary ballon. As a result, the secondary ballon exhibited slower growth. Subsequently, a tertiary ballon was generated following the same procedure as described above, resulting in a continuous flow from a macroscopic perspective. During this regime, droplet may break up from the end of the continuous flow, resulting polydisperse diamensions (Figure S11b, Supporting Information), which agrees with former studies.^[^ ^22^, ^38^ ^]^

### 2.3 Data‐Driven Predictive Model for Droplet Generation

**Figure 4:**
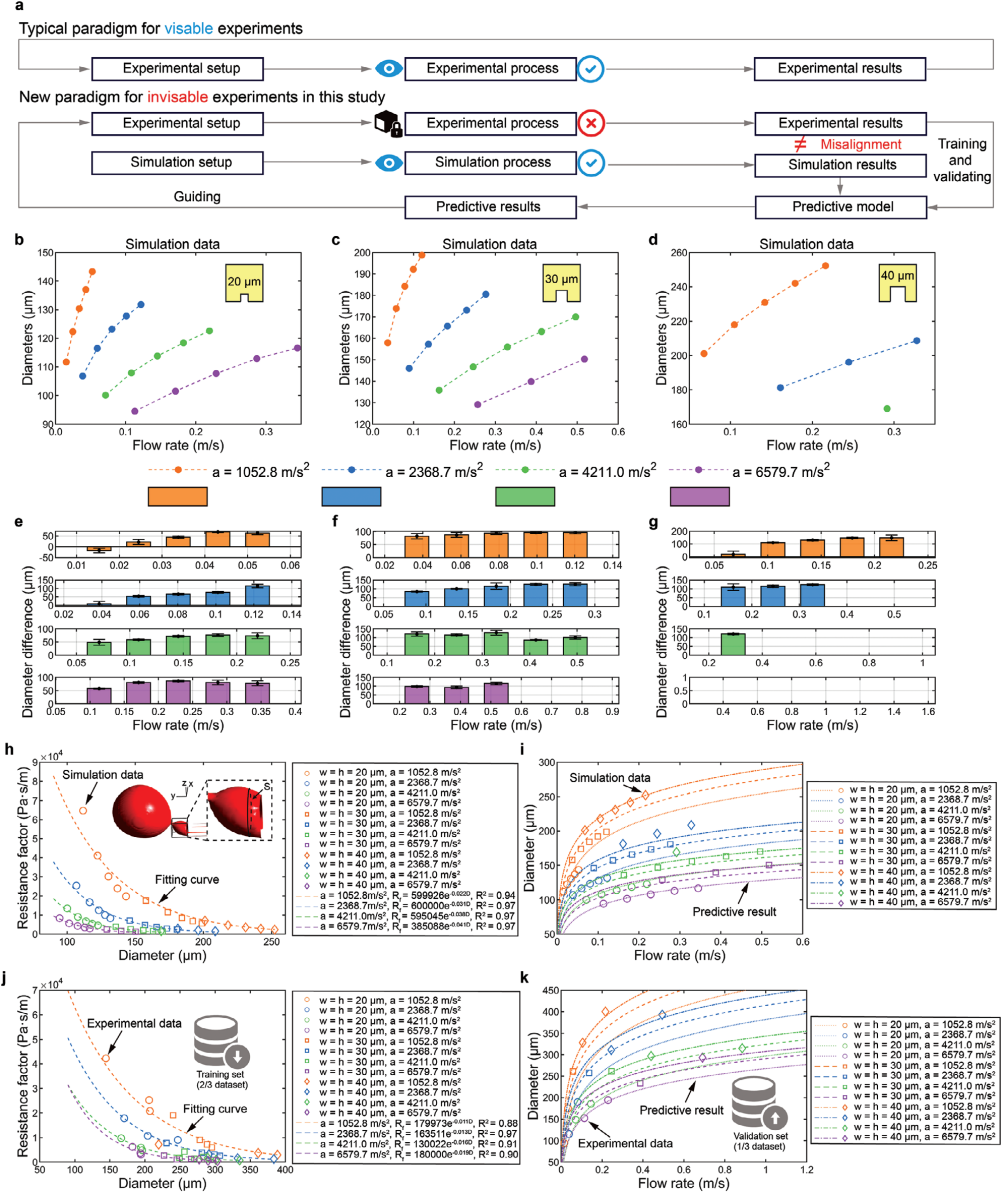
Prediction for droplet generation in the periodic dripping regime. a) Comparison of paradigms used in traditional visible experiments and the invisible experiments in this study. b) Droplet diameters generated by 20 µm microchannels (c 30 d 40 µm microchannels) through CFD simulation. e) Diameter differences between the simulation and experimental results for 20 µm microchannels (f 30 g 40 µm microchannels) (*n* > 1000, data represented as mean ± SD). h) The resistance factors of the connection tube when a droplet is about to break up against droplet diameters from the simulation dataset. The internal illustration depicts the geometry of the connection tube when a droplet is about to break up. i) The predictive results of droplet diameter based on simulation dataset under various conditions of flow rate, centrifugal acceleration, and microchannel dimensions. j) The exponential fitting through 2/3 experimental dataset to train the predictive model. k) The validation of the predictive model through the 1/3 experimental dataset with an average error rate of 4.8%.

In most visible experiments, the experimental process can be analyzed to interpret the results. However, in our invisible experiments, the CFD simulation replaced the “black box” by providing both visualization of processes and results. Despite this, idealizations in parameter design during simulation setup, coupled with the complexities of practical operations, can sometimes lead to misalignment between simulation and experimental results. To address this issue and improve the accuracy of droplet generation predictions, a new paradigm was proposed (**Figure** **4**a).

Through CFD simulation, a dataset of droplet generation under the same sixty different conditions was acquired (Figure 4b–d). Some conditions lead to droplet generation take the form of the dripping faucet, thus not labeled in Figure 4c,d. However, it is evident from Figure 2e–g that there is a misalignment between the simulation and experimental results, indicating that the simulation alone cannot accurately predict droplet generation (Figure 4e–g). Therefore, we constructed a predictive model based on geometry analysis from simulation processes and results. We assumed that when the crucial connection tube is about to break up due to Rayleigh‐Plateau instability, its geometric construction is relative to the dimensions of the ballon and microchannel. The pressure difference between the microchannel and the ballon can be calculated by combining Equations (5) and (6):

(7) $$P_{\mathrm{channel}}-\;P_{\mathrm{drop}}=2\gamma \left(\frac{1}{w}+\frac{1}{h}\right)-\;\frac{4\gamma }{D}=2\gamma \left(\frac{1}{w}+\frac{1}{h}-\frac{2}{D}\right)$$

Due to the irregular shape of the tube (illustrated internally in Figure 4h), the flow rate within the tube *V*_tube_ can be expressed according to the Young‐Laplace equation:

(8) $$V_{\mathrm{tube}}=\frac{P_{\mathrm{channel}}-P_{\mathrm{drop}}}{\int R_{\mathrm{hyd}\_\mathrm{tube}}\cdot S\;dy}$$

where $R_{\mathrm{hyd}\_\mathrm{tube}}$ corresponds to the hydraulic resistance of the tube, *S* corresponds to the cross section on the x‐z plane, and *dy* corresponds to the differentiation of the tube in the y axis direction. To simplify the calculation, we define the integral $\int R_{hyd\_tube}\cdot S\;dy$ as a resistance factor *R*_f_, and approximate *V*_tube_ with the inlet flow rate, V_in_. The resistance factor can be expressed by combining Equations (4), (7), and (8) as:

(9) $$R_{f}=\frac{2\gamma \left(\frac{1}{w}+\frac{1}{h}-\frac{2}{D}\right)}{V_{\mathrm{in}}}$$

where the diameter of approximate sphere of the ballon *D* is the parameter to be predicted, while the other parameters can be accurately calculated or measured. Once the resistance factor is determined under various conditions, it can be used to predict the final droplet diameter. To establish this relationship, simulation results were analyzed, and a scatter plot of resistance factor versus droplet diameter was constructed (Figure 4h). The data reveals that resistance factors are exponentially related to droplet diameters under the same centrifugal acceleration, irrespective of microchannel dimensions. This relationship can be expressed as:

(10) $$R_{f}=\alpha e^{-\beta D}$$

where α and β are coefficients that vary with centrifugal acceleration. The coefficients α and β were determined through data fitting and remained constant for a given centrifugal acceleration. By combining Equations (4), (9), and (10), it becomes possible to quantitatively predict droplet diameters across the flow rate interval of the periodic dripping regime, even for microchannels with varying dimensions:

(11) $$\;\frac{2\gamma \left(\frac{1}{w}+\frac{1}{h}-\frac{2}{D}\right)}{V_{\mathrm{in}}}=\alpha e^{-\beta D}$$

This predictive framework was derived from and validated against the simulation dataset, offering a robust method to tailor droplet sizes (Figure 4i) (Figure S12, Supporting Information). The corresponding generation frequencies can be also acquired according to Equation (4):

(12) $$f=\frac{whV_{\mathrm{in}}}{\frac{4}{3}\pi {\left(\frac{D}{2}\right)}^{3}}=\frac{6whV_{\mathrm{in}}}{\pi D^{3}}\;$$

We further applied the predictive model to experimental results. Specifically, 2/3 of the experimental dataset were used to train the predictive model and establish a series of exponential relationships (Figure 4j). Then the remaining 1/3 of the dataset were used to validate the predictive results, yielding an average error rate to 4.8%, which indicates accurate prediction (Figure 4k) (Figure S13 and Table S3, Supporting Information). Thus, the dataset generated by the CASE, assisted with CFD simulations, enhanced the precision of droplet generation predictions, making droplet generation pre‐design feasible.

In a broader perspective, step constructions similar to the rectangular straight‐through microchannel share the same droplet generation procedure. Therefore, the predictive model may also be suitable for them. To prove this, we applied the predictive model to a vacant rectangular microchannel and a vacant circular microchannel through simulations (Figure S14, Supporting Information). The results exhibited the same tendency in the resistance factor as the primitive microchannel (Figures S15 and S16, Supporting Information). This indicates that our predictive model is universal for centrifugal step emulsification based on straight‐through microchannels.

### 2.4 On‐Site Detection Cross Sample Scale in the CASE

The diversity of analytical samples in droplet microfluidics places higher demands on droplet platforms, particularly in terms of droplet size adjustment. The droplet pre‐design capability of the CASE meets these demands by allowing for directionally adjustment of droplet diameters from 80 to 400 µm. Before sample encapsulation, a droplet tiling operation was required for better observation of droplets stacked in light oil within the CASE. To address this, we developed three droplet tiling strategies, making the CASE a fully integrated platform for droplet pre‐design, generation, manipulation and on‐site detection (Text S1 and Figures S17–S19, Supporting Information). As a proof of concept, we successfully encapsulated samples ranging from nanoscale to microscale in droplets and conducted on‐site detection.

#### 2.4.1 Case 1: Integrated Droplet Digital Loop‐Mediated Isothermal Amplification

**Figure 5:**
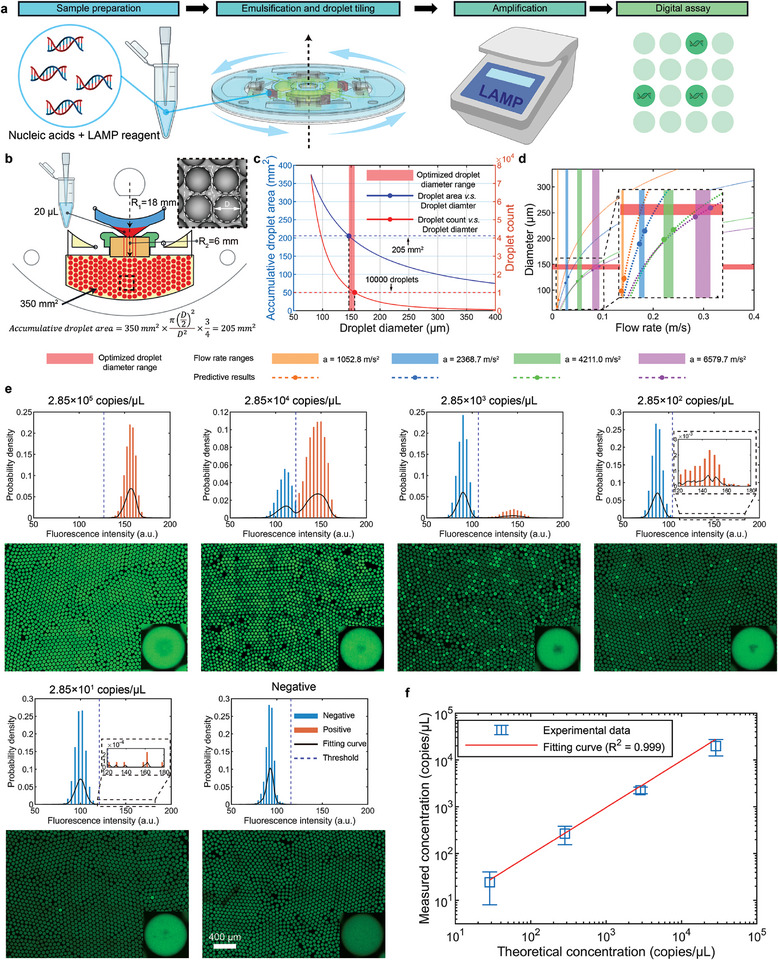
Loop‐mediated isothermal amplification in CASEs. a) The workflow of ddLAMP utilizing the CASE. b) Factors constraining droplet size such as reservoir area and spacing requirements. c) Optimization of droplet size. The blue dotted line represents an accumulate droplet area of 205 mm^2^, and the red dotted line indicates a droplet count of 10 000. d) Reverse‐engineering of generation conditions to achieve droplets within the optimized diameter range, based on predictive model results. e) The relative fluorescent intensity and corresponding images of scanned droplets with sample concentrations ranging from 2.85 × 10^1^ to 2.85 × 10^5^ copies µL^‒1^. The internal pictures depict positive droplets with marked precipitates while negative droplets don't have any. f) The linear fitting of measured nucleic acid concentrations and theoretical concentrations (*n* = 3, data represented as mean ± SD).

Nucleic acids are among the most important nanoscale biomarkers for diagnostics, typically characterized using qualitative or relatively quantitative methods such as qPCR. However, with the assistance of droplet microfluidic technology, more precise absolute quantitation is achievable.^[^ ^39^ ^]^ Herein, we employed droplet digital Loop‐mediated isothermal Amplification (ddLAMP), a sensitive and temperature‐stable nucleic acid amplification technique ideal for point‐of‐care testing,^[^ ^40^, ^41^ ^]^ to quantify the *malB* gene of Escherichia coli with the CASE (**Figure** **5**a). The sequence of the gene and its corresponding primers are provided (Table S4, Supporting Information). To initiate the LAMP reaction, we utilized a plasmid diluted into a series of test samples. A typical total mixture of 20 µL LAMP reaction reagents (Table S6, Supporting Information) was emulsified by a CASE, resulting in  *R*_2_ of 6 mm. To minimize chip size,  *R*_1_ was set to be 18 mm. The final tiled droplet size was constrained by the droplet reservoir area, measuring 350 mm^2^, to ensure the effective capture of fluorescent signals from all droplets. The spacing between droplets was also accounted for by reserving the inter‐droplet area. This was achieved by subtracting the area of droplet from a square with a side length equal to the droplet diameter, with the remaining area allocated as spacing. Additionally, one‐fourth of the total reservoir area was designated to ensure rapid droplet tiling without stacking. Consequently, the accumulated droplet area was set to 208 mm^2^ (Figure 5b). To maintain a sufficient detection dynamic range, the number of droplets was set to exceed 10 000. These conditions dictated an optimal droplet diameter range of 144 to 156 µm (Figure 5c). Using this range, the required droplet generation conditions was reverse‐engineered through our predictive model. When a 20 µm microchannel and a centrifugal acceleration of 6579.7 m s^−2^ were applied, the predicted droplet diameter range matched the target (Figure 5d). Consequently, these conditions were selected as the final parameters for droplet generation, yielding approximately 12 500 droplets with an average diameter of 145 µm. Following droplet tiling, the CASE was heated to 65 °C and incubated for fifty minutes to achieve optimal fluorescence signals during the amplification process (Figure S20, Supporting Information). Consequently, the signal of positive droplets could be distinguished from negative droplets by analyzing their relative fluorescence intensity (Figure 5b) (Figure S21, Supporting Information). In addition to fluorescent enhancement, marked precipitates resulting from the super‐saturated magnesium pyrophosphate were also observed in positive droplets, consistent with findings in published literature.^[^ ^42^, ^43^, ^44^ ^]^ As a result, *malB* gene ranging from 2.85 × 10^1^ to 2.85 × 10^4^ copies µL^−1^ was quantified through Poisson distribution model. The measured concentrations exhibited a good linear correlation with the theoretical concentrations provided by suppliers, with an *R*‐squared value of 0.999 (Figure 5c). This suggests that the CASE is capable of accurately quantifying nucleic acids. The limit of detection (LOD) for ddLAMP was determined to be 2.85 × 10^1^ copies µL^−1^.

#### 2.4.2 Case 2: Digital Bacteria Enumeration and Antibiotic Susceptibility Testing

**Figure 6:**
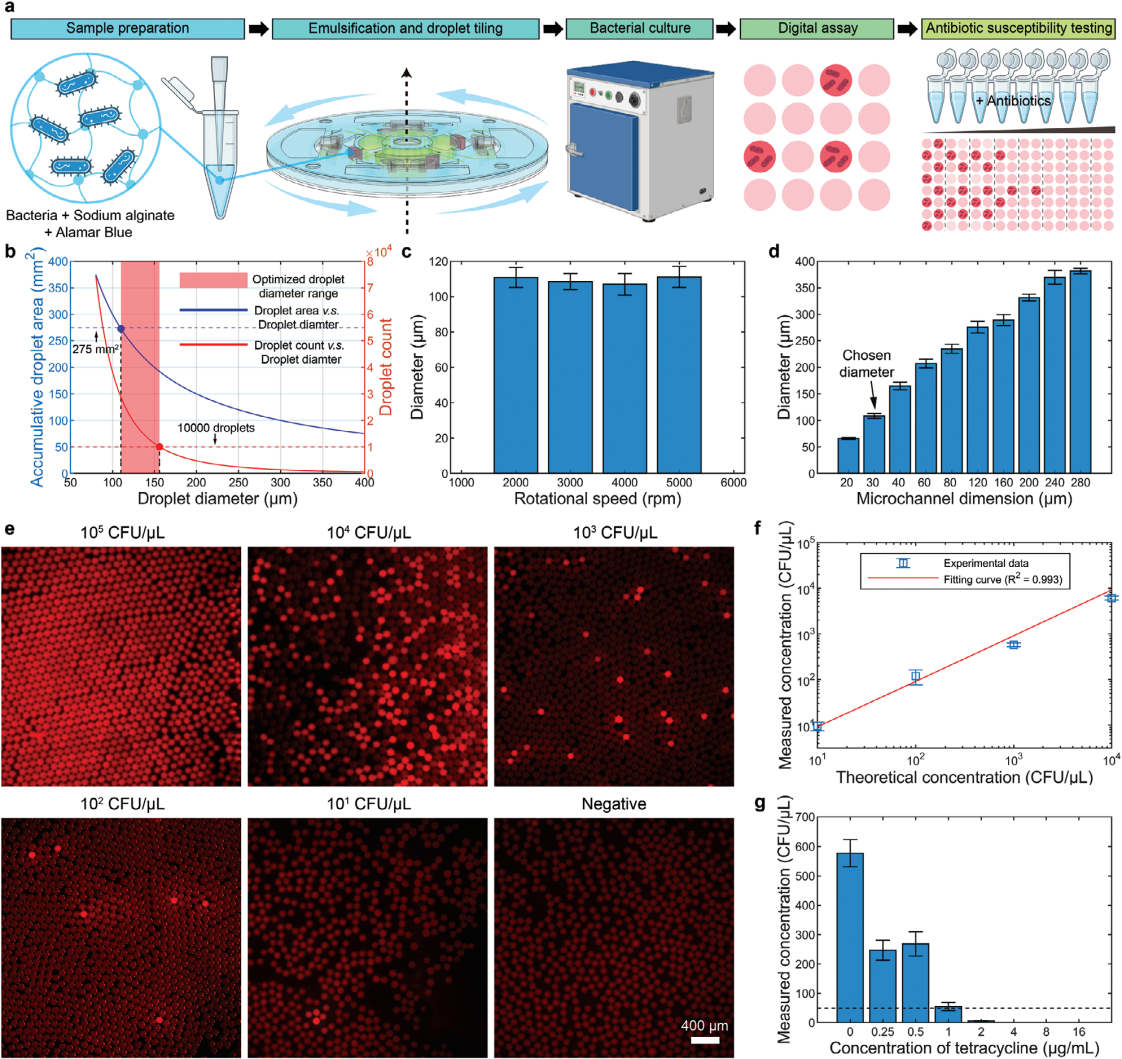
Droplet digital bacteria enumeration and antibiotic susceptibility testing (AST) by the CASE. a) The workflow of droplet digital bacterial assay and AST utilizing the CASE. b) Optimization of droplet size. The blue dotted line represents a total droplet area of 275 mm^2^, and the red dotted line indicates a droplet count of 10 000. c) Sodium alginate droplet sizes show minimal dependence on rotational speed variations (30 µm microchannels, *n* > 10 000, data represented as mean ± SD). d) Sodium alginate droplet sizes are adjustable via microchannel dimensions (*n* > 50, data represented as mean ± SD). e) Sodium alginate droplets with different bacterial concentrations ranging from 1 × 10^1^ to 1 × 10^5^ CFD µL^−1^. f) The linear fitting of measured bacterial concentrations and theoretical concentrations (*n* = 3, data represented as mean ± SD). g) Droplet digital AST of *E. coli* against tetracycline (*n* = 3, data represented as mean ± SD). The horizontal dotted line indicates the 10% survival rate.

In addition, the CASE enables the analysis of microscale bacteria. In this study, we successfully conducted droplet digital bacteria enumeration and antibiotic susceptibility testing (AST) of *E. coli* DH5α (**Figure** **6**a). To prevent bacterial sedimentation during rotation (Figure S22, Supporting Information), we employed sticky sodium alginate as the aqueous phase. Given that bacteria require prolonged incubation at the body temperature, no additional space was allocated for rapid droplet tiling. Consequently, the accumulated droplet area was set to $350\times \;\frac{\pi {\left(\frac{D}{2}\right)}^{2}}{D^{2}}=275\;mm^{2}$, reserving only the spacing between droplets. This setup indicated an optimal droplet diameter range of 110 to 156 µm (Figure 6b). However, using sodium alginate as the aqueous phase rendered droplet sizes less sensitive to variations in rotational speed (Figure 6c). This finding implied that droplet size adjustments could only be achieved by modifying the microchannel dimensions (Figure 6d) (Figure S23, Supporting Information). In practice, bacterial suspensions of varying concentrations (20 µL each) were emulsified using 30 µm microchannels, generating approximately 30 000 droplets per sample with an average diameter of 108 µm. Subsequently, droplets were tiled and put in 37 °C for ten hours of cultivation. Bacteria within the droplets are capable of reducing Alamar Blue to a red fluorescent product, thereby generating fluorescent signals. Consequently, a range of bacterial suspensions from 10^1^ to 10^4^ CFU µL^−1^ were quantified, yielding results consistent with the theoretical concentrations, achieving an *R*‐squared value of 0.993 (Figure 6e,f). The LOD for digital bacteria enumeration in the CASE was determined to be 10^1^ CFU µL^−1^. Furthermore, we assess the susceptibility of *E. coli* DH5α against tetracycline. Following the guidelines of the Clinical Laboratory Standards Institute (CLSI) and the European Committee on Antimicrobial Susceptibility Testing (EUCAST), bacterial suspensions with a concentration of 5 × 10^2^ CFU µL^−1^ were mixed with tetracycline at concentrations ranging from 0 to 16 µg mL^−1^. The observed probability of bacteria per droplet aligned with the Poisson distribution, suggesting that individual bacteria were randomly discretized within the droplet array. Accordingly, the minimum inhibitory concentration (MIC) of tetracycline against *E. coli* was eventually determined to be 1 µg mL^−1^ (Figure 6g).

#### 2.4.3 Case 3: High‐Throughput Cell Spheroid Formation and Long‐Term Cultivation

**Figure 7:**
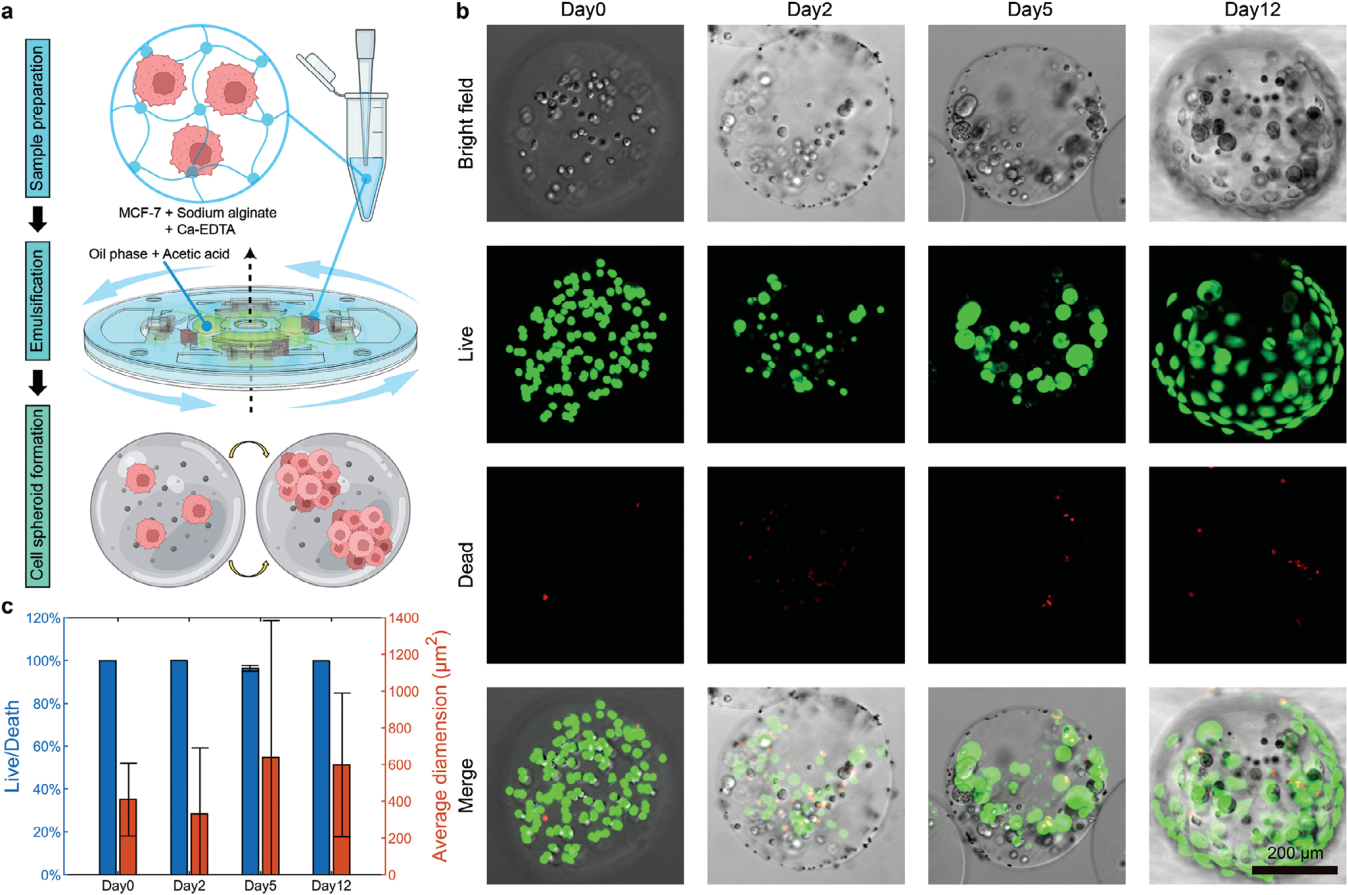
Generation of gel microspheres encapsulating MCF‐7 cells by the CASE and formation of cell spheroids. a) The workflow of gel microspheres generation utilizing the CASE and cell spheroid formation. b) The cell growth in a gel microsphere during a 12 d cultivation period. c) The ratio of live cells to dead cells and average dimension during the 12 d cultivation period (*n* = 11 for the ratio of live to dead cells; *n* = 90, 35, 39, and 71 respectively for average dimensions, data represented as mean ± SD).

Last but not least, the CASE demonstrates its capability to culture larger microscale cancer cells within gel microspheres. Breast cancer remains the most prevalent malignant tumor among women and ranks fifth among the leading causes of cancer‐related deaths worldwide.^[^ ^45^ ^]^ To meet the demands of breast cancer research, effective cultivation of breast cancer cells is essential. Calcium alginate gel microspheres, serving as a porous and solid medium, provide an optimal environment for nutrient exchange and growth support.^[^ ^46^ ^]^ Therefore, we encapsulated Michigan Cancer Foundation‐7 (MCF‐7) cells using calcium alginate droplets with CASEs (**Figure** **7**a). In particular, MCF‐7 cells were mixed with equal concentrations of sodium alginate and calcium‐ethylenediaminetetraacetic acid (Ca‐EDTA) as the liquid phase, while the primary oil phase contained 0.1% acetic acid. The Ca^2+^ ions were chelated by the EDTA without triggering a reaction with sodium alginate. However, once emulsification begins, the acidic environment in the oil phase releases the Ca^2+^ ions from the EDTA, resulting the gelation of alginate microspheres. To provide sufficient living space for cells, facilitate long‐term cultivation, and promote cell aggregation into spheroids, we employed our largest microchannel to generate the largest possible droplets (Figure 6d). Accordingly, around 1700 gel microspheres with an average diameter of 380 µm were generated using 50 µL cell suspension. Each gel microsphere encapsulated an average of 30 MCF‐7 cells. During a 12 d cultivation period, single cells within gel microspheres proliferated and condensed into cell spheroids (Figure 7b). The ratio of live cells to dead cells remained around 99.9% during cultivation, demonstrating good biocompatibility of the droplets generated by the CASE (Figure 7c). And areas of cell spheroids increased as time went by, indicated well cell proliferation and spheroid formation.

## 3 Discussion

We presented a step emulsifier that leverages customized assembled microchannels and chambers to generated monodisperse droplets in a centrifugal field. The flexibility of the CASE and the assistance of CFD simulation enabled rapid data acquisition and comprehensive geometric analysis. Based on the experimental and simulation datasets, a universal predictive model of step emulsification based on straight‐through microchannels was developed for tailored droplet generation. This made droplet pre‐design, generation, manipulation and on‐site detection integrate in a single chip to satisfy biochemical assays cross sample scale in droplet microfluidics.

The fabrication of desired 3D microchannels and chambers in the CASE complies with typical assemble of discrete elements in modular microfluidics.^[^ ^47^, ^48^, ^49^, ^50^ ^]^ The combination of soft lithography technology and laser engraving technology integrates the advantages of both, providing delicate microstructures and centimeter rigid chambers. Such a flexible combination offers a new method for rapid 3D microfluidic manufacturing even though sacrificing some precision for manual assembly. However, although the current CASE is based on some common materials and techniques used in the laboratory, the total manufacturing process is still too complicated for automated production. Furthermore, the manually assembled CASE brings uncertainty to the geometric construction, which may lead to polydisperse droplets especially when the microchannel is extremely small in size. Therefore, a more precise, rapid prototyping and flexible 3D microfluidic manufacturing technology is needed for future work.

We also demonstrated that CASE can be used to pre‐design and generate desirable droplets through a data‐driven predictive model. Through a large set of data generated by the CASE and simulation, the resistance factor of a crucial connection tube was found to finally determined droplet size, which can be used to predict droplet generation at a wide range of microchannel dimensions, flow rates and centrifugal accelerations. Accordingly, users can choose the droplet size and generation frequency as wanted, avoiding extensive validation experiments for droplet generation. Compared to conventional regression‐based scaling laws that rely solely on experimental data, our CFD‐integrated framework allows visualization and in‐depth analysis of droplet formation dynamics, leading to the identification of key process parameter. Furthermore, CFD simulations provide extensive datasets across diverse conditions, mitigating underfitting risks associated with limited experimental data. This CFD‐experiment integrated approach not only strengthens the robustness of the predictive model but also showcases its adaptability for forecasting droplet generation in other systems with similar structural configurations. Thus, our framework demonstrates broader applicability and reliability, bridging the gap between theoretical modeling and practical implementation. Moreover, our predictive model outperforms prior methods in predictive range and applicability (**Table** **1**). Currently, our predictive model was only verified by the straight‐through microchannel. However, there are various step orifice structures exhibit minor differences in construction but yield significantly different performances in droplet generation for step emulsification, thus a more universal model is required for centrifugal step emulsification. Additionally, except from utilizing geometric models, droplet size prediction based on machine learning is also a good strategy which has not been applied in step emulsification to our knowledge.^[^ ^20^, ^21^, ^51^ ^]^ Through the flexible and rapid prototyping CASE, a bigger dataset of droplet generation can be acquired to train and validate machine learning models.

**Table 1:** Comparison of droplet prediction methods in existing works and the CASE framework.

References	Step orifice structures	Actuator	Predictive diameter range of droplet [µm]	Predictive equation	Principle	Predictive performance
CASE	Straight‐through microchannel	Centrifuge	80–400	$\;\frac{2\gamma (\frac{1}{w}+\frac{1}{h}-\frac{2}{D})}{V_{\mathrm{in}}}=\alpha e^{-\beta D}$	Geometry analysis and data fitting	The average error rate is 4.8%
[35]	Straight‐through microchannel	Centrifuge	136–190	$R=\sqrt{\alpha^{\prime }\Delta p_{\mathrm{app}}+\beta^{\prime }}$	Buoyancy force equals the viscous Stokes drag after droplet breakup	Limited to the regime where bond and capillary numbers are very small
[19]	Terrace	Centrifuge	60–100	$d=\sqrt[{3}]{\frac{6V}{\pi }}\;\approx \sqrt[{3}]{\frac{6}{\pi }\left(V_{\mathrm{ct}}-V_{\mathrm{rd}}\right)}$	Geometry analysis	The relative droplet size error less than 4%
[32]	Triangular nozzle	Pump	/	$d\approx \frac{h}{\cos(\pi -\alpha )}$	Geometry analysis and energy balance approach	Valid only at low capillary numbers, where droplet size is controlled by the contact angle independent of flow rate
[52]	Co‐flow + straight through microchannel	Pump	/	$\;\frac{d}{b}=3{\left[1+\frac{\left(1+k\right)}{w/b}\right]}^{-1}$	Energy balance approach	Predicts the minimum droplet size
[53]	Terrace	Pump	10–90	$V=H[\frac{L^{2}\varphi }{4}-\frac{L(L-2A)}{4}\sin\varphi ]$	Geometry analysis	Mean percentage deviation is 4.6%

Furthermore, the integration of the three strategies for droplet tiling in CASE facilitates automated droplet microfluidics. Meanwhile, the fundamental principles of these strategies offer guidance to other macroscopic manipulation of droplets in a centrifugal field. Unfortunately, human intervention is indispensable in this study, introducing an additional destabilizing factor, which can be further avoided by integrating external equipment.

Finally, the CASE exhibits good performance in on‐site detection cross sample scale. Specifically, in the ddLAMP application, the wide and rigid droplet reservoirs of the CASE can stably store droplets, preventing fusion during heating. Additionally, the airtight PMMA chamber inhibits water evaporation, ensuring the activity of the amplifying enzyme. The presence of marked precipitates found in positive droplets can aid in determining negative and positive signals, which means excluding consumption and effects of fluorescent dyes. The digital bacterial enumeration and AST delivered rapid and accurate results at single‐cell level, demonstrating the CASE as a powerful tool for bacterial research. Additionally, the successful formation of cell spheroids and their long‐term cultivation highlight its excellent biocompatibility. Its potential in constructing 3D cell aggregates can attribute to various applications such as drug screening and intercellular interaction studies. Taken together, these applications represent the CASE as a powerful centrifugal platform for droplet microfluidics.

## 4 Experimental Section

### Fabrication and Assembly of the CASE

The chamber and microchannel patterns of both the PMMA and PDMS subassemblies were designed by AutoCAD software (Autodesk, California, USA) or SolidWorks (SolidWorks, MA, USA). Every layer of the PMMA chip and positioner was produced by a CO_2_ laser cutting (Laser Technology, China), except the layer with concentric inclined planes was fabricated by the CNC manufacturing. Layer to layer adhesion was through a pressure sensitive adhesive and the alignment was conducted by the alignment holes. The bottom of the PMMA subassembly is a layer of glass (Guluo Glass, China) with a positioning structure cut in the center, which was also adhered to the upper layer. The bonding between layers was confirmed through a thermo‐compression bond by a hot press device (Hengwei Precision Technology, China) at 50 °C for 5 min. The mold of the PDMS subassembly was made from SU8 3025 (Kakyku Advanced Materials, MA, USA) and GM 1075 (Gersteltec Engineering Solutions, Swiss) by a photolithography machine (G‐25XA, Xinnanguang, China), and the long microchannels were acquired by pouring the mixture of PDMS (Dow, NY, USA) and its curing agents (10:1) to the mold. The long microchannels were cut into 5 × 10 mm pieces by a cutting die with a 10 × 1 array. The microchannels were treated with MesoPhobic‐2000 (MesoBiosystem, China) and subsequently dried in a 65 °C oven for 2 h to ensure hydrophobicity. The PDMS and PMMA subassemblies were treated with O_2_ plasma by the PDC‐MG (Weike Spectrum, China) for 1 min and put in 65 °C for 2 h to ensure the bonding. The clearances among the air, the PDMS and PMMA subassemblies were stuffed with a sticky UV curing adhesive (D‐3322, Kafuter, China), which was UV cured soon after laying.

### Image Identification of the Droplet Size

The 2D spliced images of droplets in the horizontal plane were acquired with a 10× lens by a spinning disk confocal super resolution microscope (SpinSR10, Olympus, Japan). The original images were processed manually to remove adulterations and enhance the contrast by Adobe Photoshop (Adobe Systems, CA, USA). The droplets’ diameters were identified by a MATLAB (MathWorks, MA, USA) script. Specifically, the coordinates and radii of droplets were distinguished by imfindcircles function from Image Processing Toolbox. Complete and accurate results were ensured by adjusting properties: Sensitivity and EdgeThreshold.

### CFD Simulation Setup

All the simulations were completed by ANSYS (ANSYS, PA, USA) in the HPC Platform of Huazhong University of Science and Technology. The geometry was designed using Spaceclaim software. The meshes were generated from Meshing software by MultiZone. Condition settings and computations were performed by FLUENT software. A pressure‐based, double‐precision CFD solver was employed for transient calculation. The laminar VOF model with a velocity inlet, pressure outlet, and No Slip wall condition was utilized. For solution methods, a SIMPLE scheme was employed for pressure‐velocity coupling, a least squares cell‐based gradient, a PRESTO! pressure, a second order upwind momentum, and a Geo‐Reconstruct volume fraction were chosen for spatial discretization. For initialization, a region covering half of the microchannel starting from the inlet was patched as water, while other cells were designated as oil. All the numerical parameters used in the case are summarized (Table S2, Supporting Information).

### Droplet Digital Loop‐Mediated Isothermal Amplification

The sequence of the *malB* gene and its corresponding primers are provided (Table S4, Supporting Information). The plasmid and three pairs of primers were synthesized by Sangon Biotech (China). The LAMP primers were premixed in a certain proportion and diluted to 10× before mixing with LAMP reagents (Table S5, Supporting Information). The total reaction components are tabulated (Table S6, Supporting Information). To determine the optimal amplification time, the reaction system was incubated at 65 °C in a StepOnePlus real‐time PCR system (Life Technologies, CA, USA) while recording real‐time fluorescence signals. For ddLAMP, a gene amplifier (Avist, China) was employed to heat the CASEs after emulsification and droplet tiling. The fluorescent image of the entire droplet reservoir was scanned using confocal microscopy, and the relative fluorescence intensity of all the droplets was analyzed using Fiji software (National Institutes of Health, USA). The concentration of the target samples can be quantified through the Poisson distribution of nucleic acids in droplets:

(13) $$C=\frac{-\ln\left(1-p\right)}{V}\;$$

where *p* corresponds to the positive rate of all droplets, and *V* corresponds to the volume of each individual droplet.

### Droplet Digital Bacteria Enumeration and AST

*E. coli* DH5α bacterial cells were cultured on Luria‐Bertani (LB, Solarbio, China) agar plates. Individual colonies were selected and cultured in Cation‐Adjusted Mueller Hinton II Broth (MH II, Solarbio, China) at 37 °C overnight to generate bacterial suspensions. The liquid phase consisted of 1% w/w sodium alginate (Aladdin, CA, USA) in MH II medium with 1:10 v/v Alamar Blue (Yeasen, China). Prior to mixing with the bacterial solution, the liquid phase was filtered using a syringe‐driven filter (Biofil, China) with 0.22 µm pores to prevent contamination and microchannel blockage. After emulsification and droplet tiling, CASEs were incubated at 37 °C for 10 h. Dark field images of the droplets were captured using a CCD microscope (Olympus Corporation, Japan), and their signals were analyzed utilizing a segmentation plugin in Fiji. Positive and negative droplets in the images were manually marked to train a classifier, which was then used to automatically classify other uncategorized droplets. Finally, the positive rate of all droplets was determined, and concentrations were calculated using Poisson distribution, similar to that in ddLAMP. For AST, the tetracycline (Macklin, China) was dissolved and diluted to 1 mg mL^−1^. After filtration, it was diluted in sodium alginate solution to concentrations ranging from 0 to 16 µg mL^−1^. Thereafter, 5 × 10^2^ CFU µL^−1^ bacterial suspensions were inoculated to culture medium with tetracycline and incubated at 37 °C overnight.

### Encapsulation of MCF‐7 Cells and Cultivation

The MCF‐7 cells were cultured at 37 °C and 5% CO_2_ in Dulbecco's modified Eagle medium (DMEM, Gibco, CA, USA) supplemented with 10% fetal bovine serum (FBS, Every Green, Tianhang, China) and 1% penicillin‐streptomycin (Gibco, CA, USA). Cells was split with 0.25% trypsin/EDTA (Gibco, CA, USA) and resuspended with DMEM. Sodium alginate was dissolved in phosphate‐buffered saline (PBS) to prepare a 2.5% w/w solution, which was then mixed with the cell suspension at a ratio of 4:1 in order to obtain an inoculum of 10^6^ cells mL^−1^. A 50 mm Ca‐EDTA was prepared by mixing 100 mm disodium‐EDTA (Aladdin, CA, USA) solution and 100 mm calcium chloride (Macklin, China) solution in a 1:1 ratio, and the pH of the Ca‐EDTA was adjusted to 7.4. Finally, the aqueous phase was prepared by mixing the sodium alginate and Ca‐EDTA solutions in equal ratios. After emulsification, gel microspheres were aspirated from the CASEs and transferred to DMEM to wash away the oil phase. The MCF‐7 cells were then cultured in 24‐well plates with the culture medium being exchanged every 2 days.

### Statistical Analysis

All calculations and statistical analyses were performed using MATLAB. The consistency among continuous variables was assessed through one‐way ANOVA. Results are expressed as mean ± standard deviation (SD) based on at least triplicate measurements. The LOD was defined as the lowest concentration of samples that consistently yielded positive results in all replicate experiments. To minimize statistical error in the LAMP application, the variation in droplet fluorescence intensity due to changes in the optimal focal plane at different positions were corrected to a consistent level. The fluorescence intensity threshold for distinguishing positive from negative droplets was initially defined as the mean fluorescence intensity of negative droplets from negative sample plus three times the SD. Subsequently, the thresholds for samples with varying concentrations were adjusted based on the initial threshold to maintain consistent criteria. To address periodic fluctuations in droplet fluorescence intensity caused by image stitching, a partial judgment was applied to classify droplets as positive or negative. Droplets were classified as positive if their fluorescence intensity exceeded the mean fluorescence intensity of the surrounding ten negative droplets plus five times their SD.

## Conflict of Interest

The authors declare no conflict of interest.

## Author Contributions

X.W. and X.C. contributed equally to this work. P.C., B.‐F.L., and Y.L. conceptualized the study, guided experimental design, analyzed data, revised manuscript, and provided financial support. X.W. conceptualized the study, performed major experiments, simulation and manuscript writing. X.C. involved in chip optimization, data analysis and article proofreading. C.W. and H.Y. analyzed the data. S.L., Y.Z., R.Z., and Y.Q. involved in experimental design.

## Supporting information



Supporting Information

## Data Availability

The data that support the findings of this study are available from the corresponding author upon reasonable request.
