# Supplementary material for: Data‐Driven Theoretical Modeling of Centrifugal Step Emulsification and Its Application in Comprehensive Multiscale Analysis
Source: Adv Sci (Weinh). 2025 Feb 8;12(13):2411459. doi: 10.1002/advs.202411459 (PMC11967796; doi:10.1002/advs.202411459)
Supplement: Supplementary file 1 — Supporting Information [file ADVS-12-2411459-s001.docx]

# Supporting Information

# Data-Driven Theoretical Modeling of Centrifugal Step Emulsification and Its Application in Comprehensive Multi-Scale Analysis

*Xin Wang§, Xiaolu Cai§, Chao Wan, Huijuan Yuan, Shunji Li, Yiwei Zhang, Ran Zhao,* *Yuxi Qin, Yiwei Li*, Bi-Feng Liu*, Peng Chen**

The Key Laboratory for Biomedical Photonics of MOE at Wuhan National Laboratory for Optoelectronics-Hubei Bioinformatics & Molecular Imaging Key Laboratory, Systems Biology Theme, Department of Biomedical Engineering, College of Life Science and Technology, Huazhong University of Science and Technology, Wuhan 430074, China

§ These authors contributed equally to this work

* Corresponding authors

E-mail: [gwchenpeng@mail.hust.edu.cn](mailto:gwchenpeng@mail.hust.edu.cn)(Lead contact); bfliu@mail.hust.edu.cn; yiweili@hust.edu.cn

Tel: +86-27-87792203

Fax: +86-27-87792170

This file includes

Figures S1–S23

Tables S1–S6

Text S1

Supporting References.

## Supporting Figures:


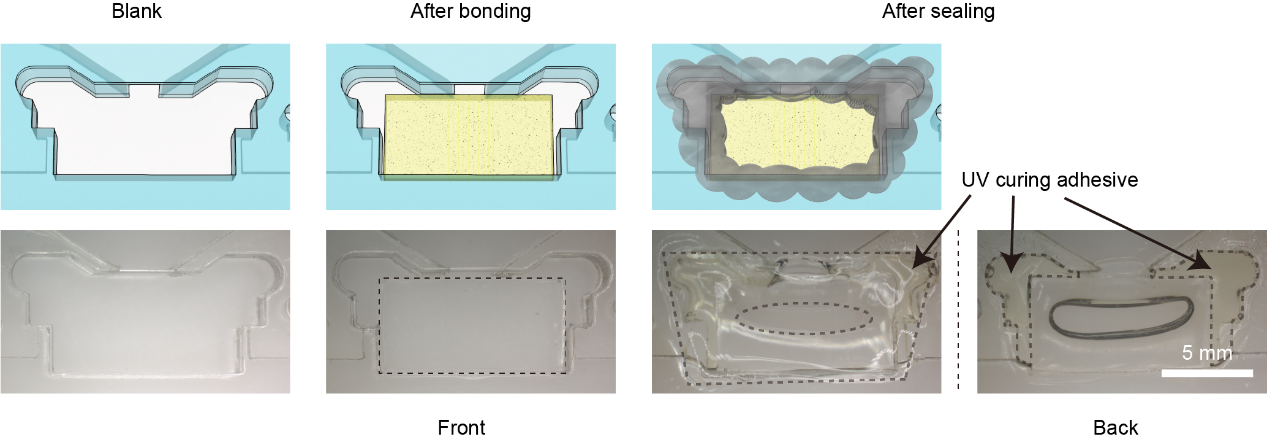


**Figure S1: The bonding and sealing processes of a PMMA and PDMS subassemblies.** First, the bottom glass of the PMMA subassembly and the PDMS subassembly were bonded together using plasma treatment. Next, clearances connected to the air were filled with a UV curing adhesive to prevent air ingress. This ensured that the aqueous phase can only enter the droplet reservoir through the microchannels.


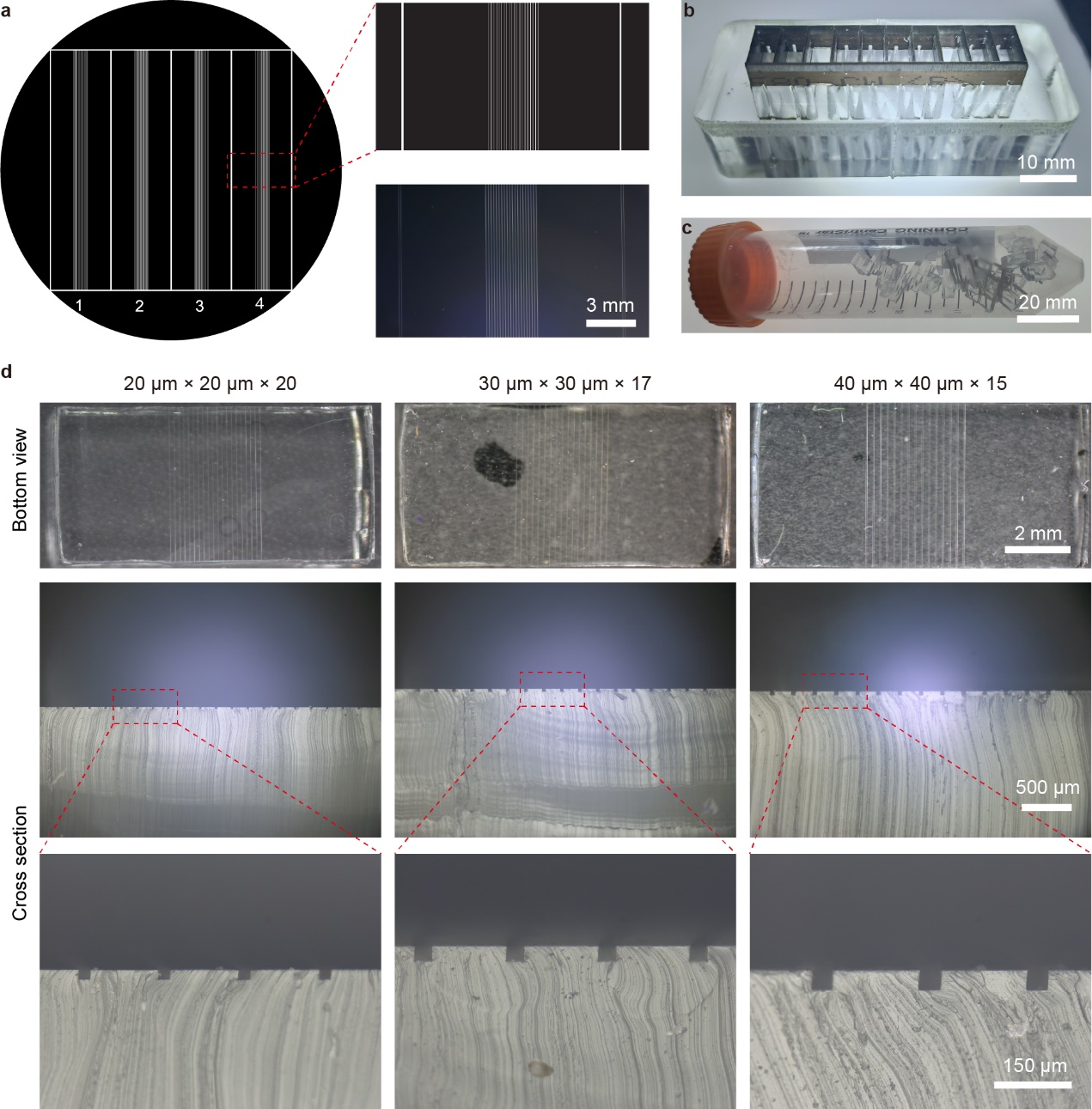


**Figure S2: High-throughput production of the PDMS subassemblies with multiple microchannel dimensions. a.** A mask of microchannels and its corresponding SU8 positive mold (bottom-right). **b.** The cutting die with an array of ten 5 × 10 mm rectangles. **c.** The mass-produced PDMS subassemblies. **d.** Bottom views and cross sections of different microchannels ranging from 20 µm to 40 µm.


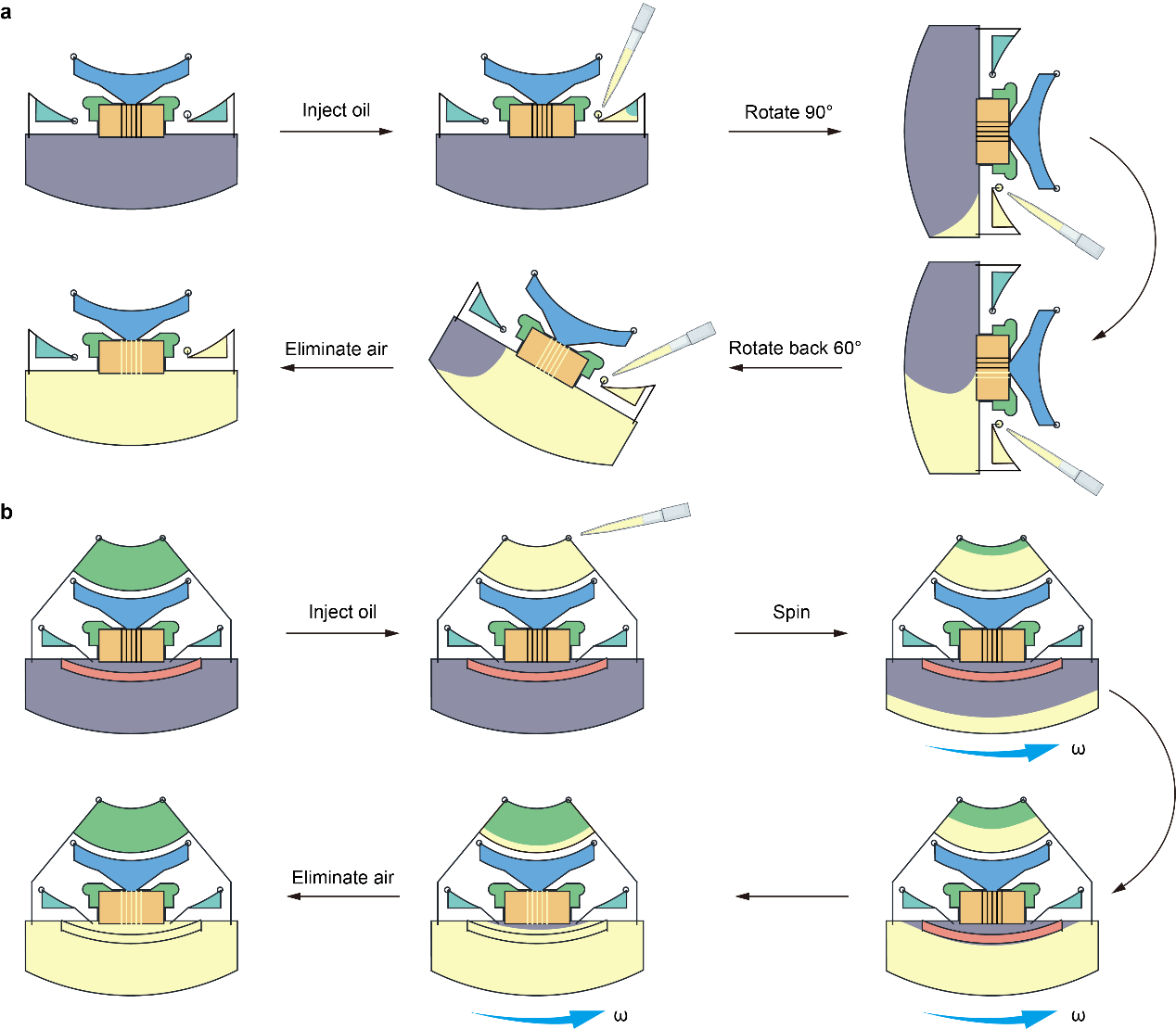


**Figure S3: Oil injection procedures for the CASE to eliminate air in the droplet reservoir. a.** A total of 300 μL of light oil phase was introduced into the droplet reservoir through the oil inlet using a 1 mL pipette tip vertically. The inlet was designed to be larger than the pipette tip's end, ensuring a secure seal during the injection process. By adjusting the position of the CASE so that the air phase was above the oil phase, air was effectively expelled. The oil injection method of the CASE utilizing the inward strategy mirrors that of the inverting strategy (Text S1, Supporting Information). **b.** The oil injection process of the CASE using the outward strategy.


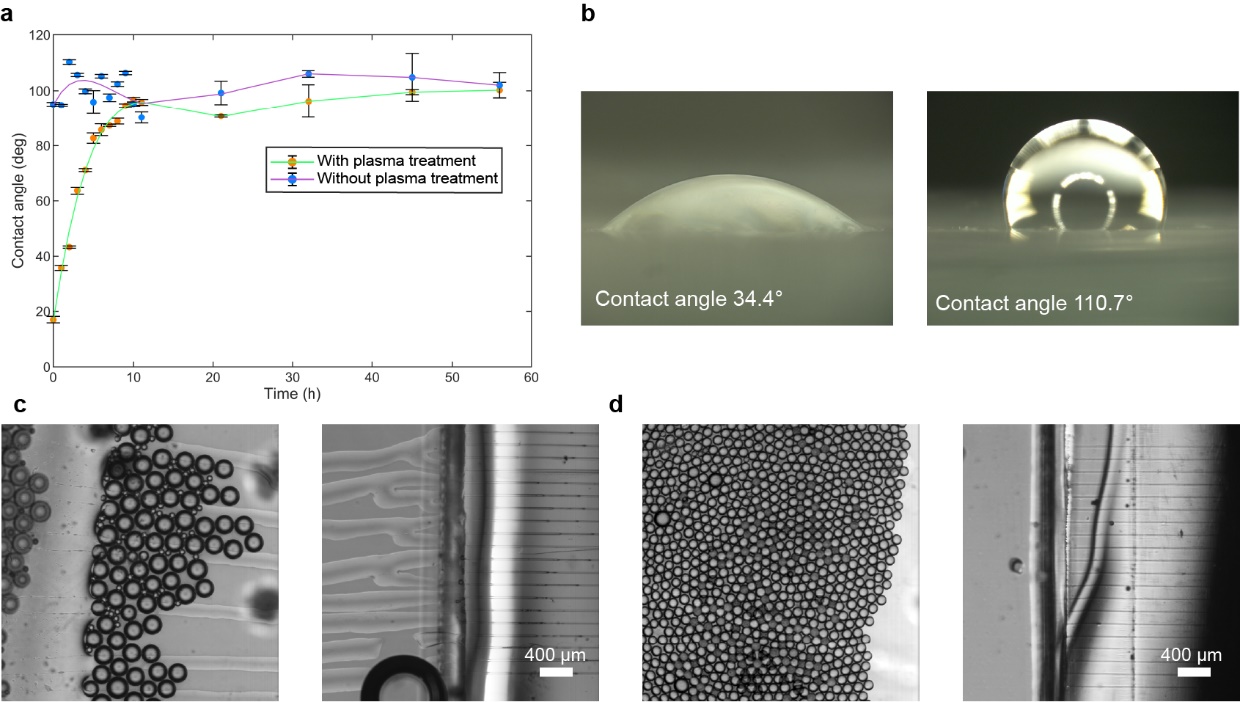


**Figure S4: The influence of wettability of microchannels to the droplet generation.** **a.** The contact angle of the PDMS surface after the plasma treatment (n = 3, data represented as mean ± SD). **b.** A droplet in PDMS surface that didn’t recover its hydrophobicity (left) and a droplet in PDMS surface that recovered. **c.** The emulsification by hydrophilic channels, whose liquid phase was adhered to the bottom glass. **d.** The emulsification by hydrophobic channels whose droplets was more monodisperse and smaller than that in **c**.


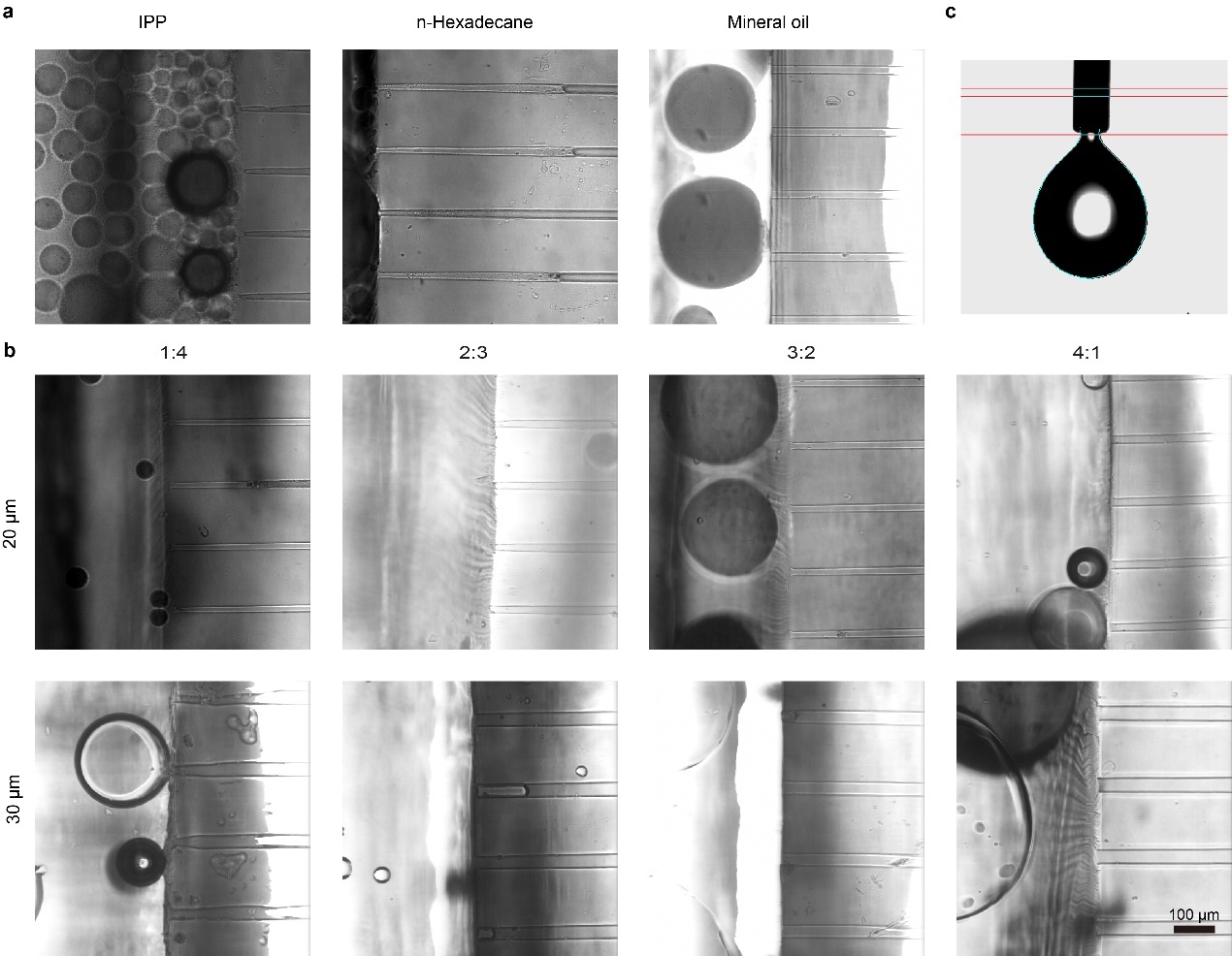


**Figure S5: Optimization of the oil phase.** **a.** PDMS microchannels exhibit varying responses to three types of oil in terms of channel collapse. Isopropyl palmitate (IPP) may cause serious collapse of microchannels, following by n-Hexadecane. However, mineral oil does not have any influence. **b.** The nozzles of microchannels showed nice integrity during emulsification when the volume ratio of the n-Hexadecane to mineral oil over 3:2. Scale bar: 400 μm. **c.** The surface tension measurement of deionized water in the optimal oil phase, which is consisted of 60% mineral oil and 40% n-Hexadecane, along with 8% v/v EM180 as surfactant.


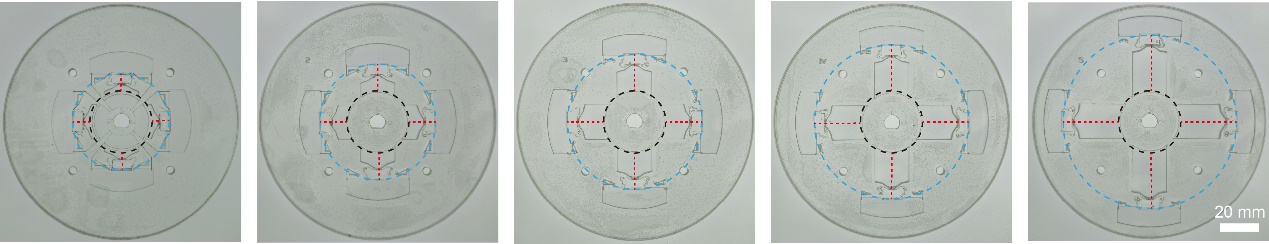


**Figure S6:** **The geometries of the PMMA subassemblies employed to droplet generation under different conditions.** The PMMA subassemblies exhibit increasing $R_{2}$ (detailed value is show in Supplementary Table S2). The red dotted lines represent $R_{2}$ for individual microfluidic chips, while the blue dotted circles depict concentric circles where the nozzles are located, and the black dotted circles depict the surface of aqueous phase whose radius is $R_{1}$.


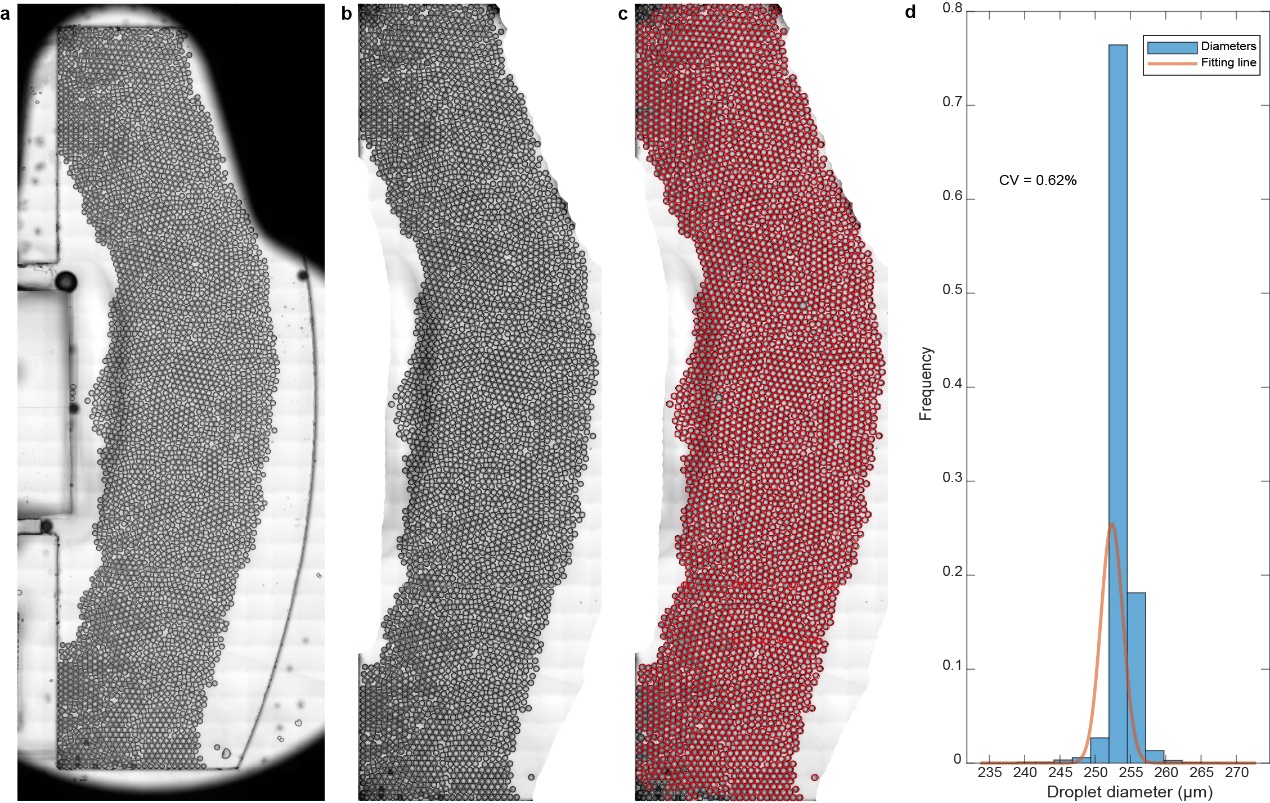


**Figure S7: Droplet diameter identification.** **a.** An original stitched bright field image of droplets by confocal. **b.** The manually processed image whose adulterations were moved and contrast was enhanced. **c.** The droplets were identified and marked by red circles. d. Histogram of the frequency distribution of droplet diameters.


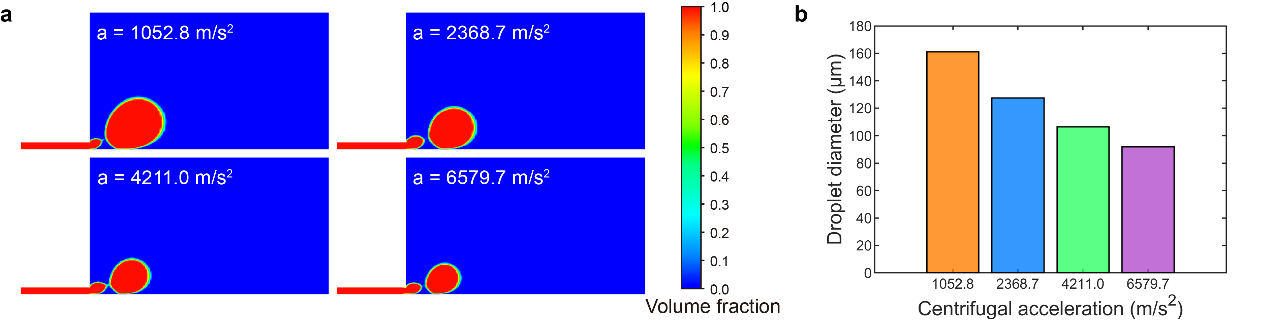


**Figure S8. The influence of centrifugal acceleration on droplet size as simulated by CFD. a.** The breakup moment of a droplet at varying centrifugal accelerations under a flow rate of 0.1 m/s in a 20 μm microchannel. **b.** Droplet diameter as a function of centrifugal acceleration. Droplet size decreased with increasing centrifugal acceleration due to higher centrifugal forces accelerating the ballon velocity, causing earlier breakup.


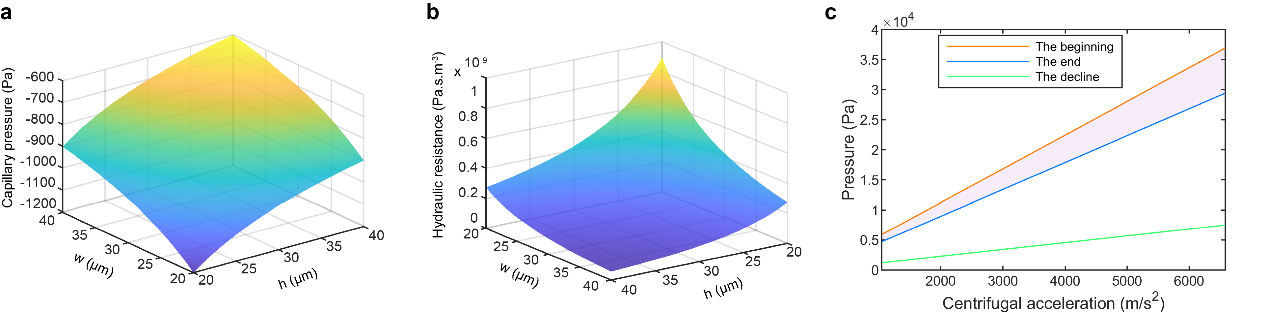


**Figure S9: Detail numerical calculations of the capillary pressure, hydraulic resistance and driving pressure.** The capillary pressure (**a**) and hydraulic resistance (**b**) against multi-sized microchannels. **c.** The driving pressure against centrifugal acceleration when the aqueous phase was 20 μL.


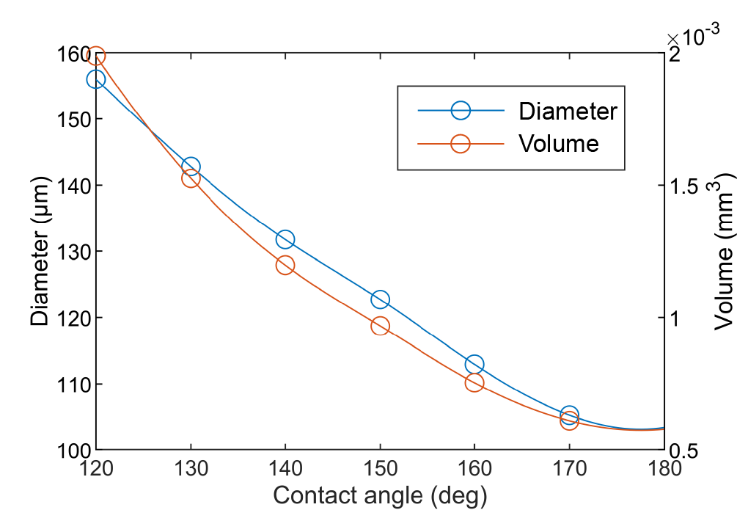


**Figure S10: The droplet diameter and volume against contact angle of the walls.** The droplet dimension decreases as the contact angle increases, indicating that a lower contact angle setup corresponds to more calculation.


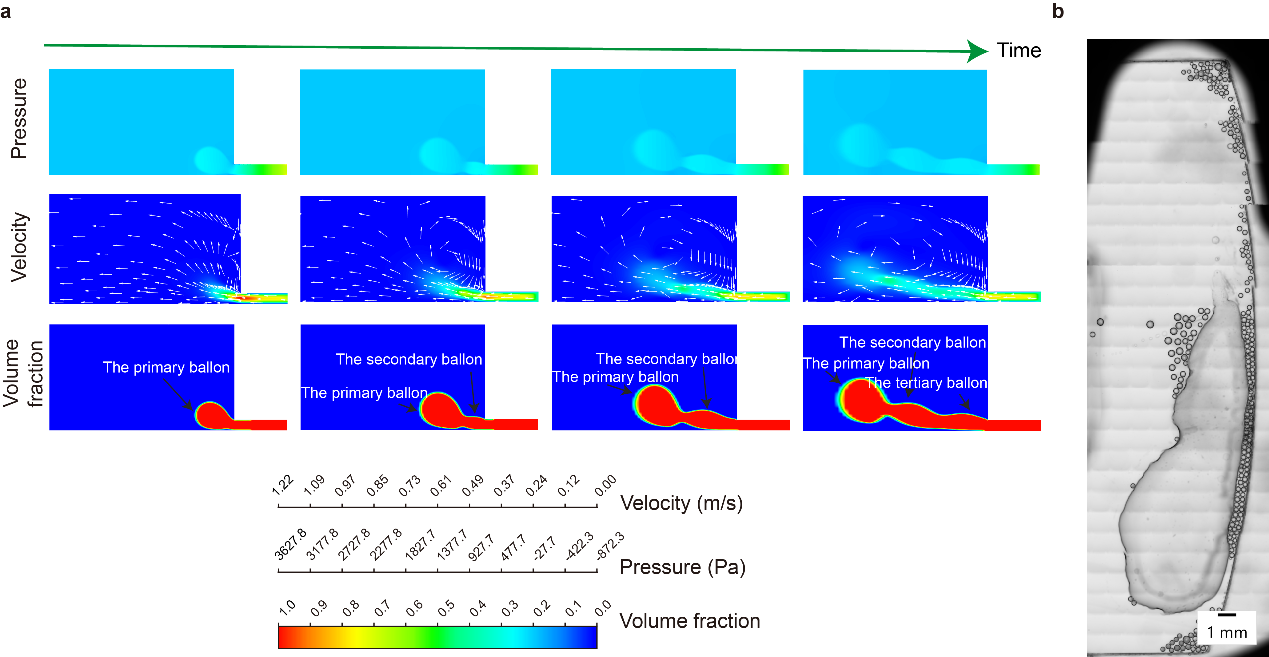


**Figure S11: Droplet generation in the regime of dripping faucet. a.** Numerical simulation of the procedure of droplet generation in the regime of dripping faucet. Initially, a primary balloon formed due to the instantaneous relief of geometric constraints in the step. However, the velocity enhancement resulting from the Laplace pressure difference was negligible compared to the swift flow rate in the microchannel. Consequently, the shrinking of the connection tube was insufficient to cause breakup due to Rayleigh-Plateau instability. Furthermore, a secondary balloon formed, connecting the primary balloon and the microchannel, followed by a tertiary balloon and so forth. From a macroscopic perspective, this process resulted in a continuous flow. **b.** An end-point image of emulsification in the dripping faucet mode ($w=h=40 \mu m, a=6579.7 m/s, V_{in}=1.39 m/s$).


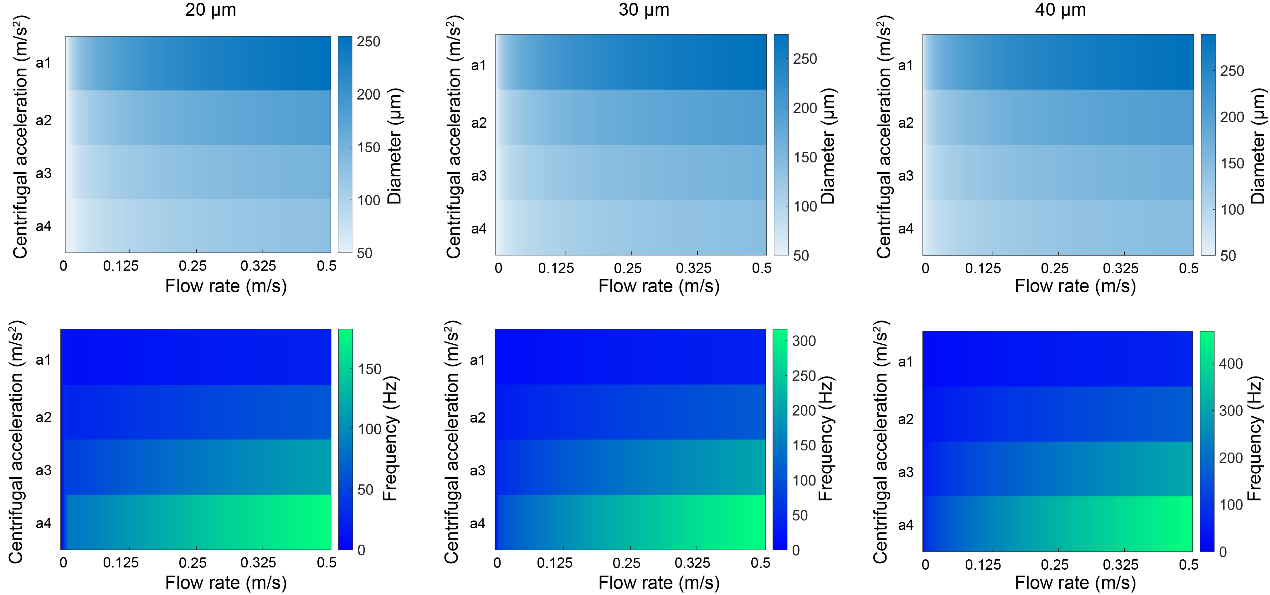


**Figure S12: The prediction of droplet dimensions and generation frequencies through CFD simulation.** a1 to a4 refer to centrifugal accelerations ranging from 1052.8 to 6579.7 m s^-2^.


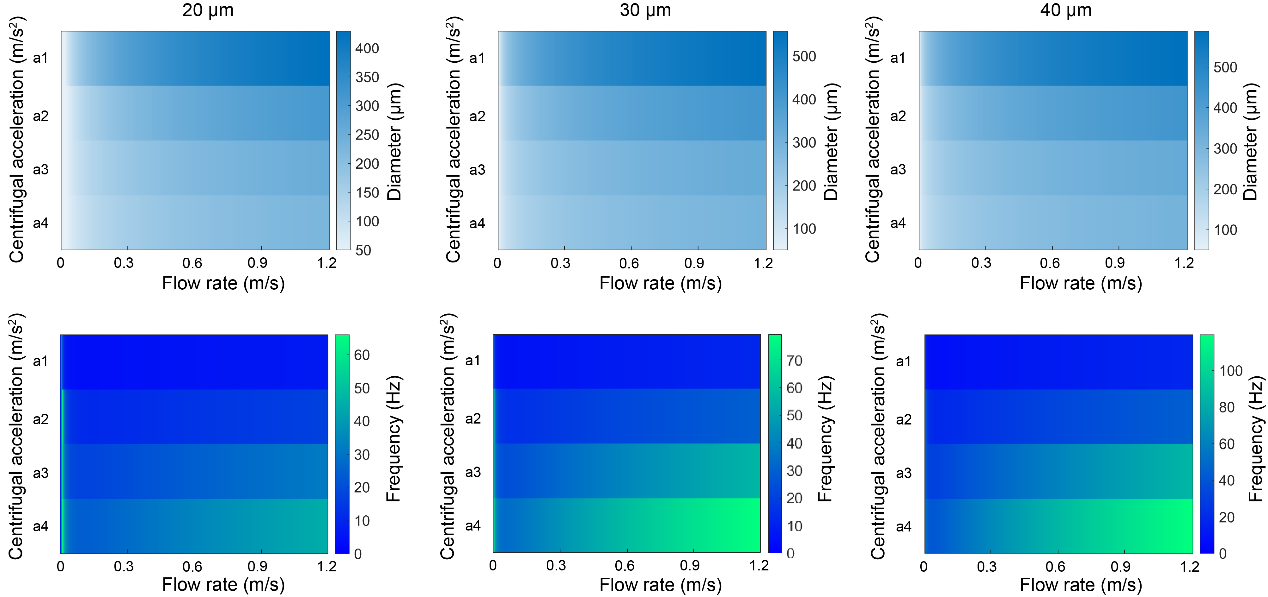


**Figure S13: The prediction of droplet dimensions and generation frequencies through the known experimental results.**


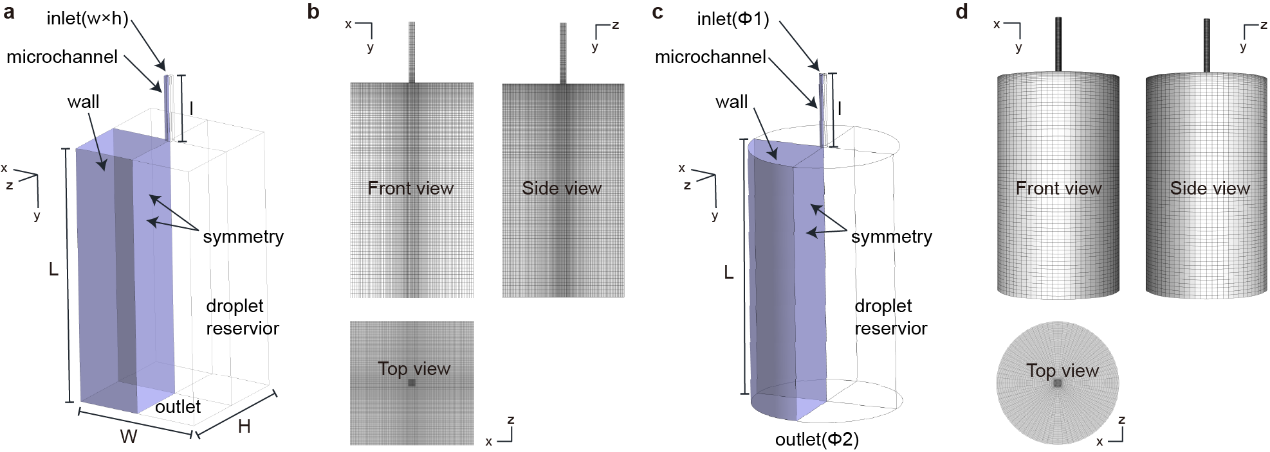


**Figure S14: The three-dimensional models and structured grid partitions for the vacant rectangular microchannel (a. b.) and the vacant circular microchannel (c. d.).**


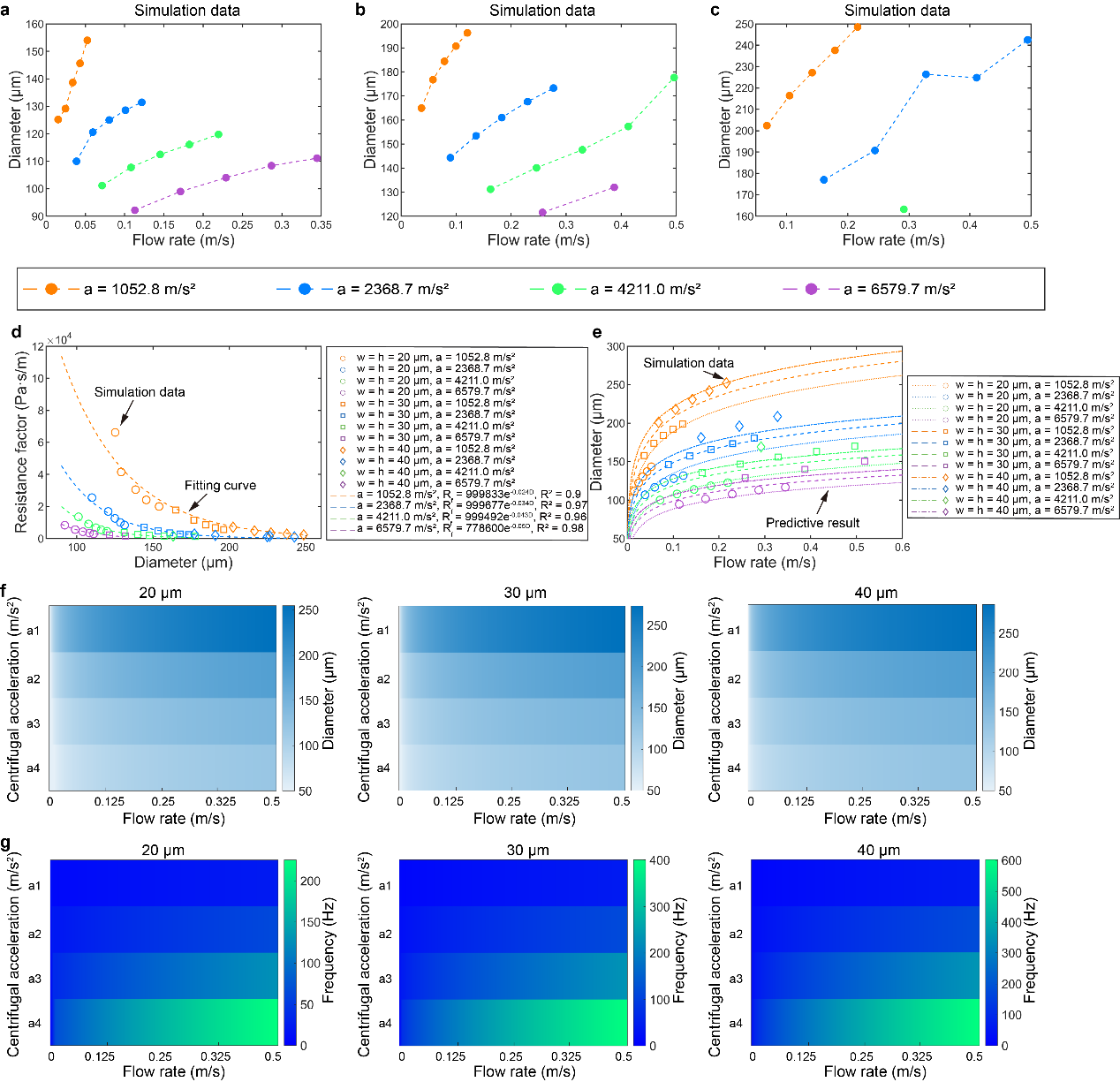


**Figure S15: The predication results for the vacant rectangular microchannel.** Droplet diameters against $V_{in}$ generated by **a.** 20 **b.** 30 **c.** 40 μm microchannels. **d.** The resistance factors of the connection tube when a droplet is about to break up against droplet diameters. **e.** The prediction of droplet diameters based on the resistance factors (in the form of a heatmap in **f.**). **g.** The prediction of droplet frequencies based on the resistance factors.


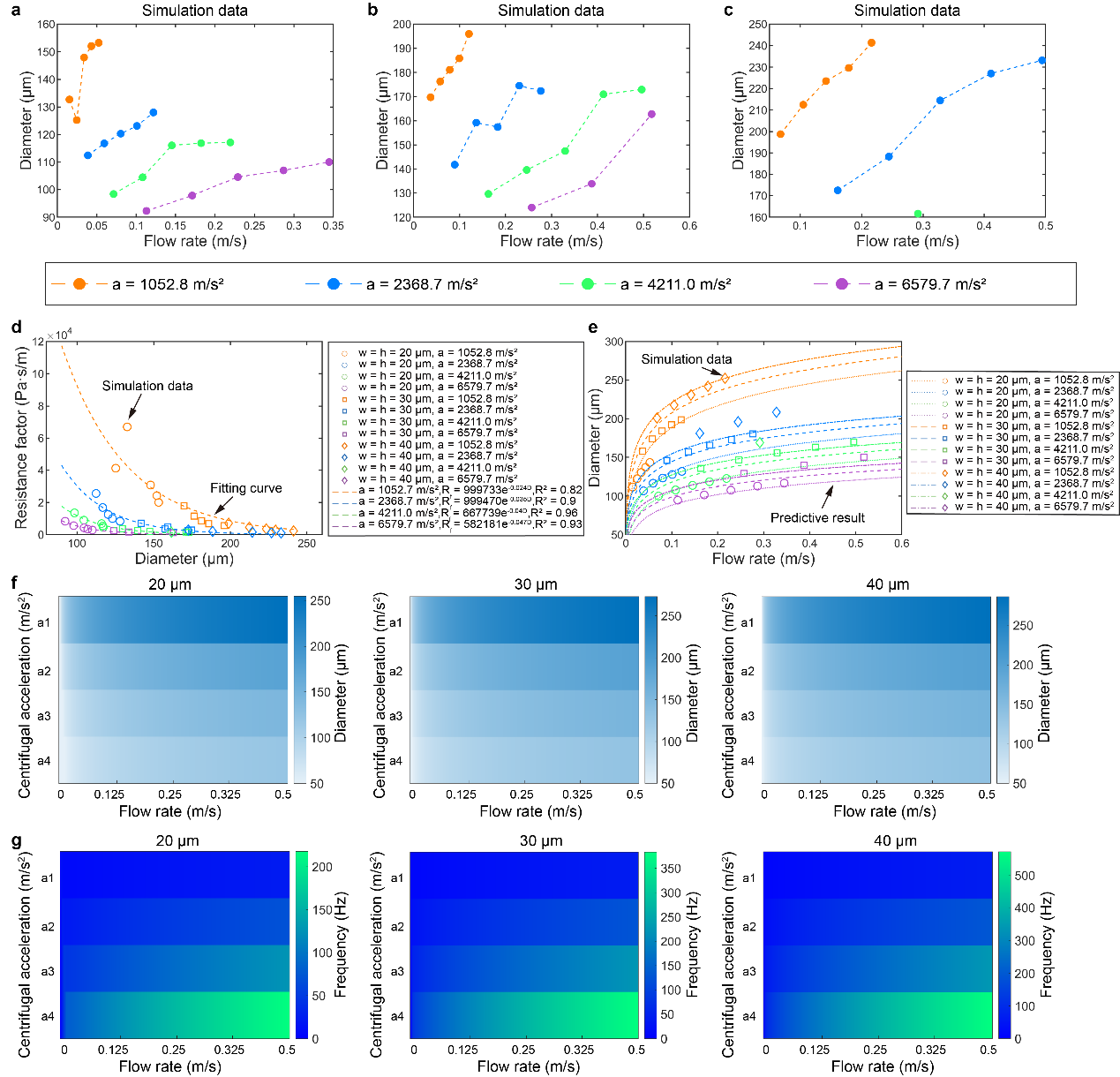


**Figure S16: The predication results for the** **vacant circular microchannel.** Droplet diameters against $V_{in}$ generated by **a.** 20 **b.** 30 **c.** 40 μm microchannels. **d.** The resistance factors of the connection tube when a droplet is about to break up against droplet diameters. **e.** The prediction of droplet diameters based on the resistance factors (in the form of a heatmap in **f.**). **g.** The prediction of droplet frequencies based on the resistance factors.


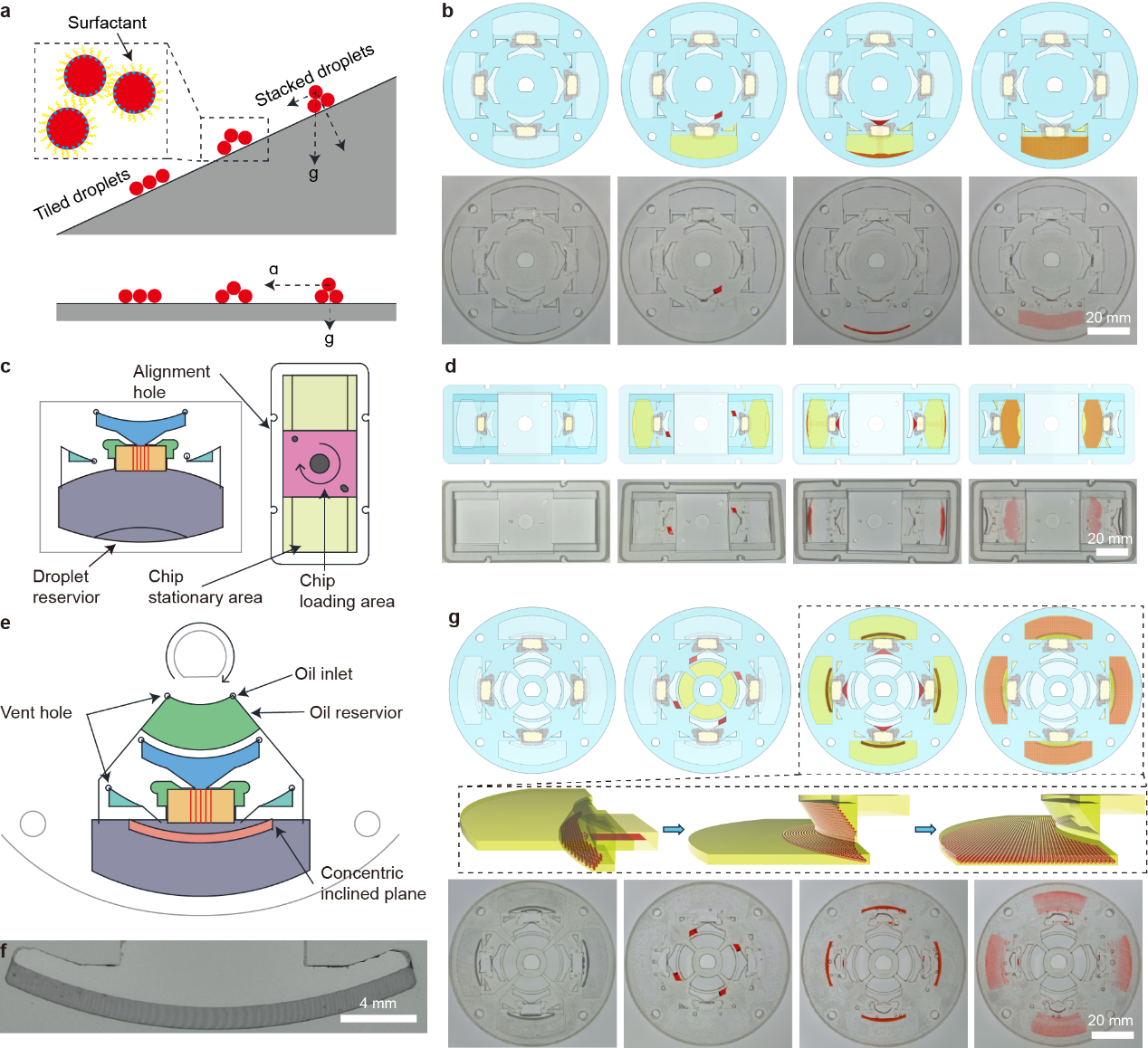


**Figure S17: Three strategies for droplet tiling in the CASE. a.** Schematic of droplet tiling strategies by gravity and centrifugal force. The horizontal component of gravity or the centrifugal force drives droplets to a broader area, while the vertical component of gravity or the gravity in the centrifugal filed drives upper stacked droplets down to the surface. **b.** The procedure of the inverting strategy. **c.** The functional structures of a CASE utilizing the inward strategy and its positioner. **d.** The procedure of the inward strategy. **e.** The functional structures of a CASE utilizing the outward strategy. **f.** A concrete figure of the concentric inclined plane. **g.** The procedure of the outward strategy.


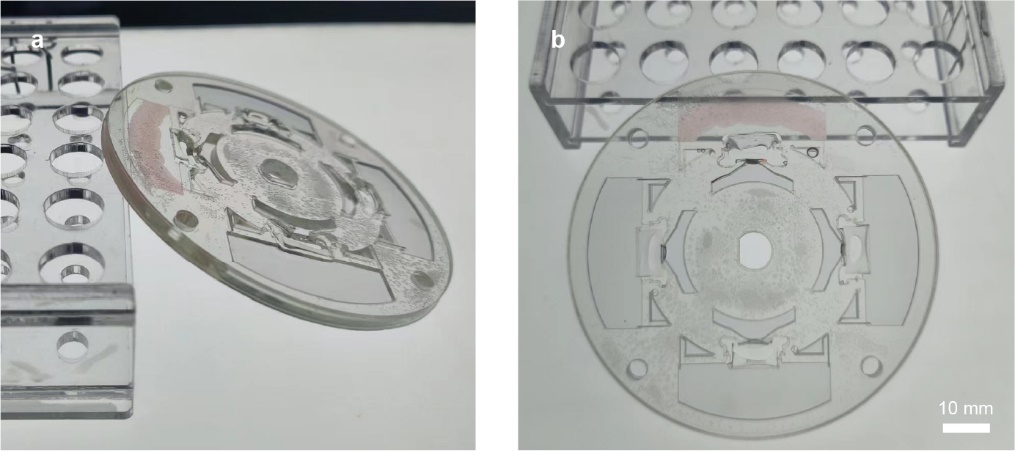


**Figure S18: Droplet tiling utilizing the inverting strategy. a.** Side view and **b.** Top view of droplet tiling in a CASE using the inverting strategy. Scale bar:10 mm.


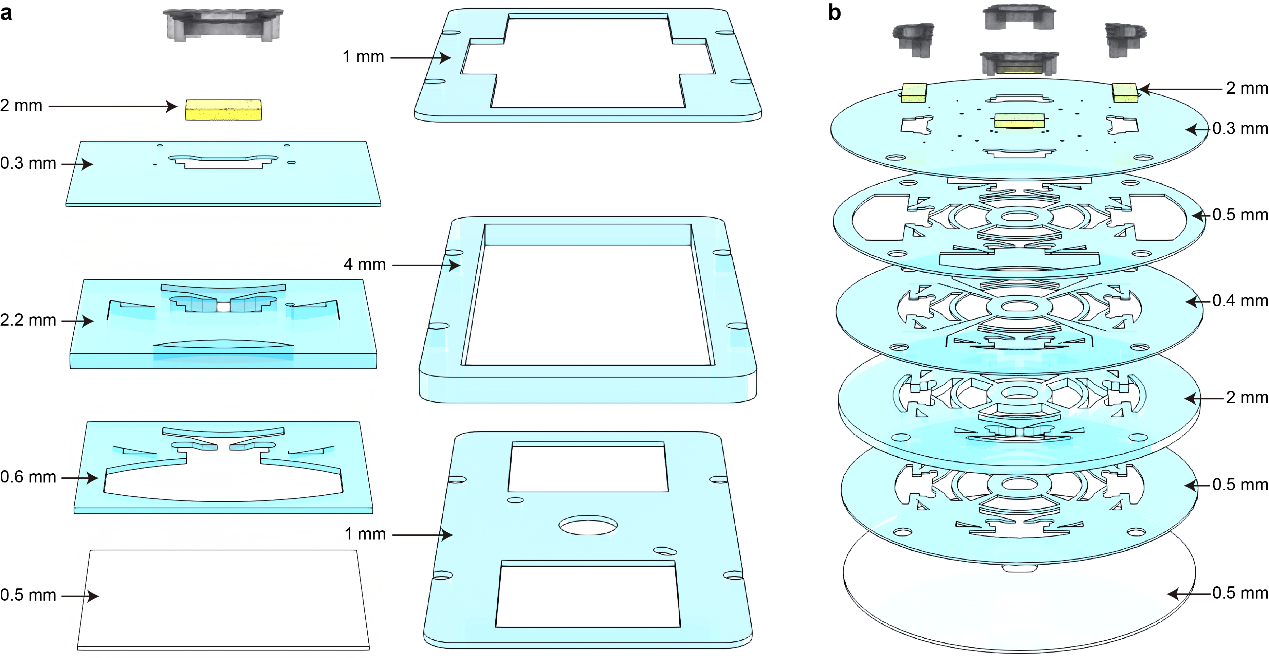


**Figure S19: The exploded views of microfluidic chips utilizing the inward and outward strategy.** a. The cartridge chip with a CASE (left) and the positioner (right). b. The microfluidic disk used for outward strategy.


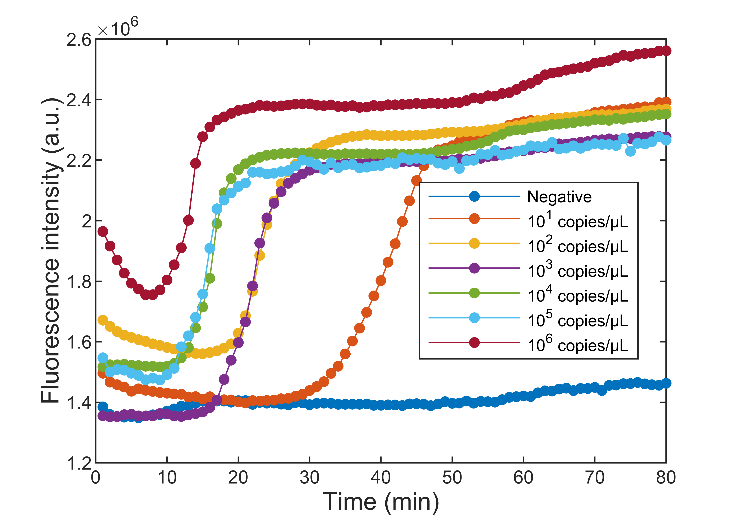


**Figure S20: Optimization of amplification time.** 50 min was chosen as the optimal time.


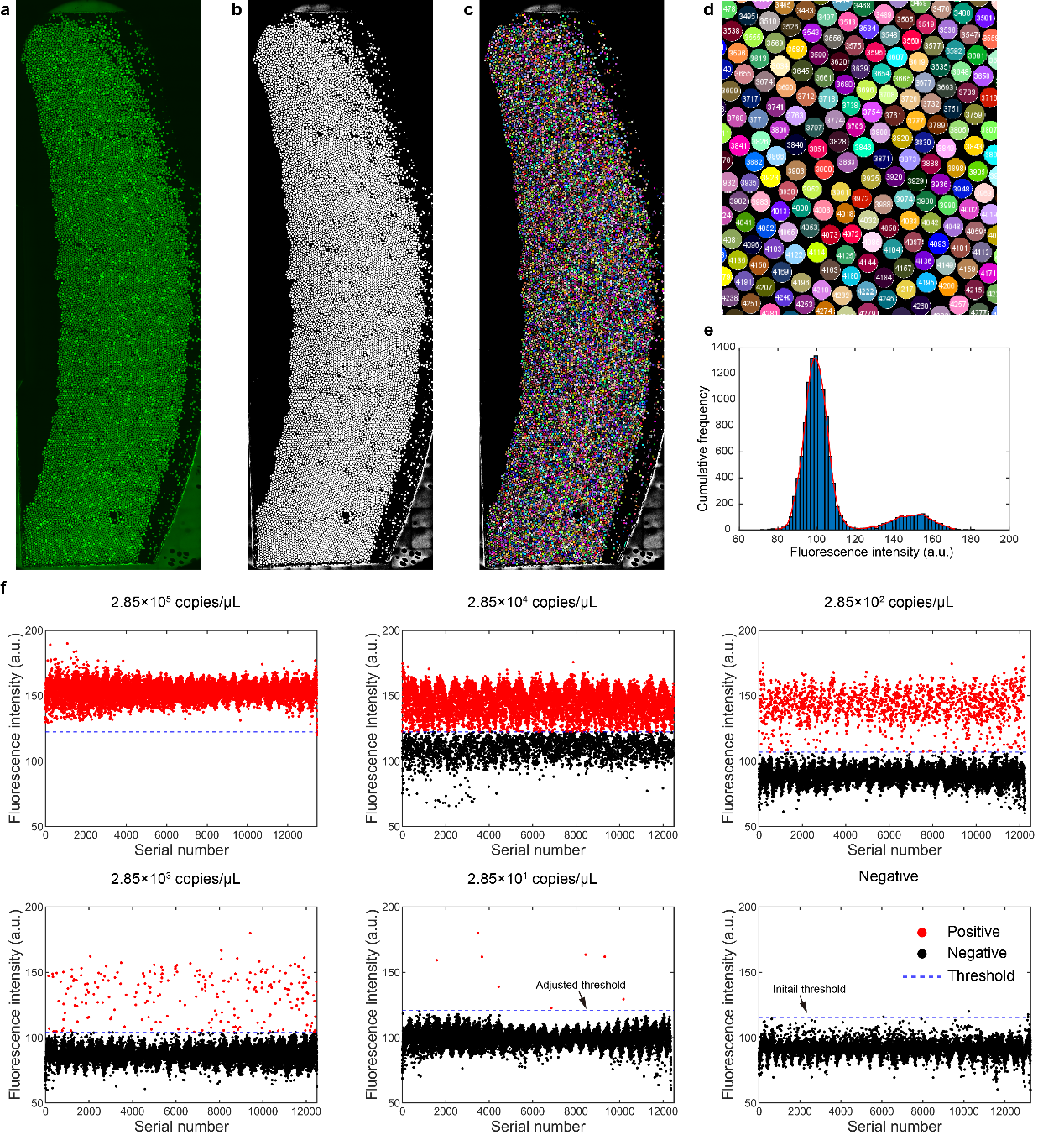


**Figure S21: Differentiation of negative and positive signals by imageJ. a.** An original fluorescent image of droplets. **b.** Fluorescent areas of droplets were distinguished from the dark background and marked in white. **c.** Individual droplets were identified using Analyze Particles. **d.** A detailed image from **c**, with each fluorescent droplet numbered. **e.** Distribution of fluorescence intensity among all droplets. **f.** Scatter plots of droplets with sample concentrations ranging from 2.85 × 10^1^ to 2.85 × 10^5^ copies/µL.


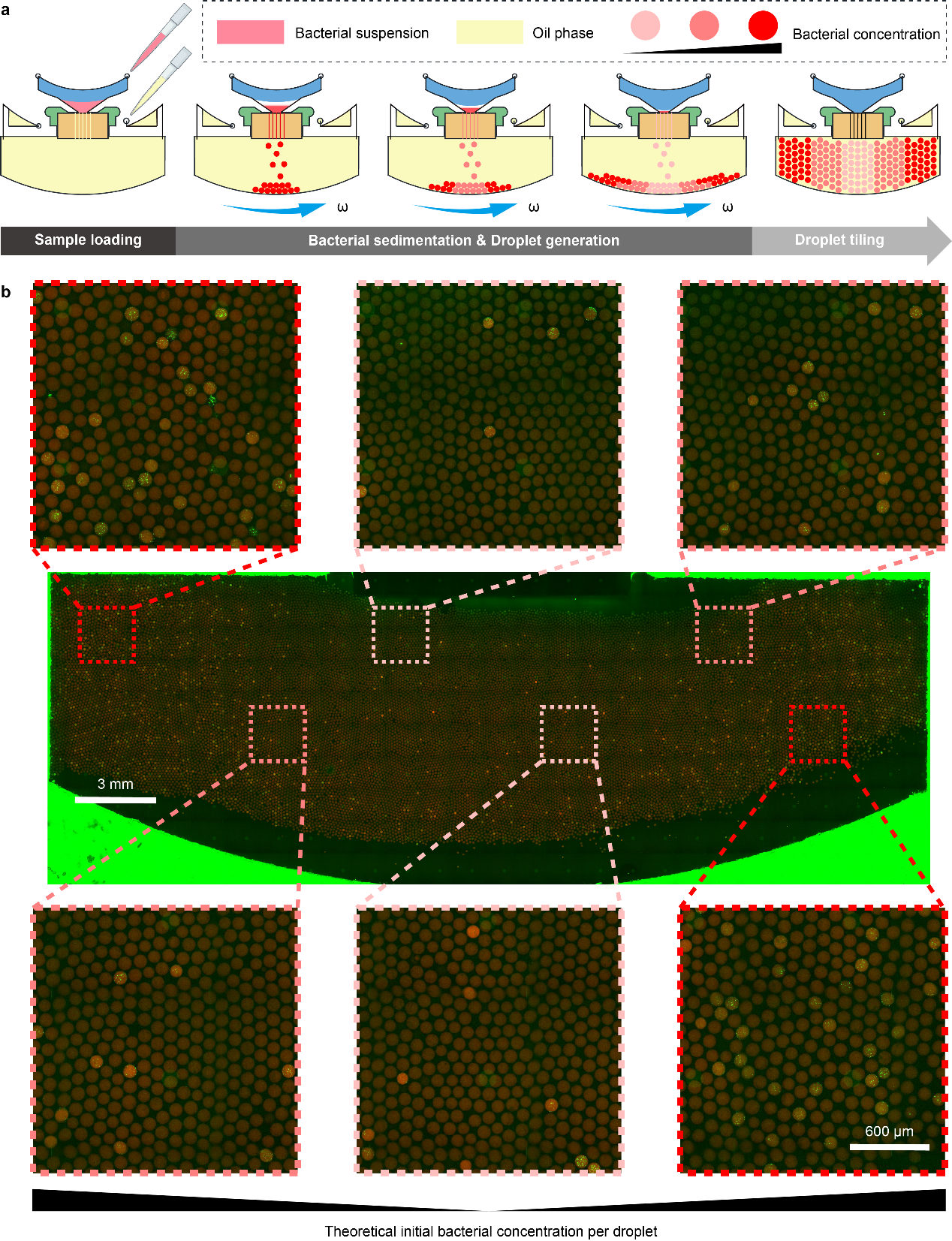


**Figure S22: Bacterial sedimentation in bacterial culture medium during rotation induces space variation in theoretical initial concentrations per droplet. a.** Schematic illustration of the process leading to spatial variation. In the sample loading stage, bacteria are evenly distributed in the suspension. As rotation commences, bacterial sedimentation occurs within the suspension due to density differences between bacteria and the aqueous phase, while droplets are generated and deposited into the droplet reservoir. Droplets formed later displace those generated earlier, pushing them towards the edges of the droplet reservoir. Consequently, once the droplets tile, the theoretical initial bacterial concentration exhibits a gradient, decreasing progressively from the sides to the center of the droplet reservoir. **b.** Experimental validation of spatial variation in theoretical initial concentrations. *E. coli* producing green fluorescent protein were cultured in MH II medium for 10 hours with 1:10 v/v Alamar Blue (red fluorescence) as an indicator. Consistent with the theoretical model, the initial bacterial concentration decreases from the edges towards the center of the droplet reservoir, as observed through fluorescence signals.


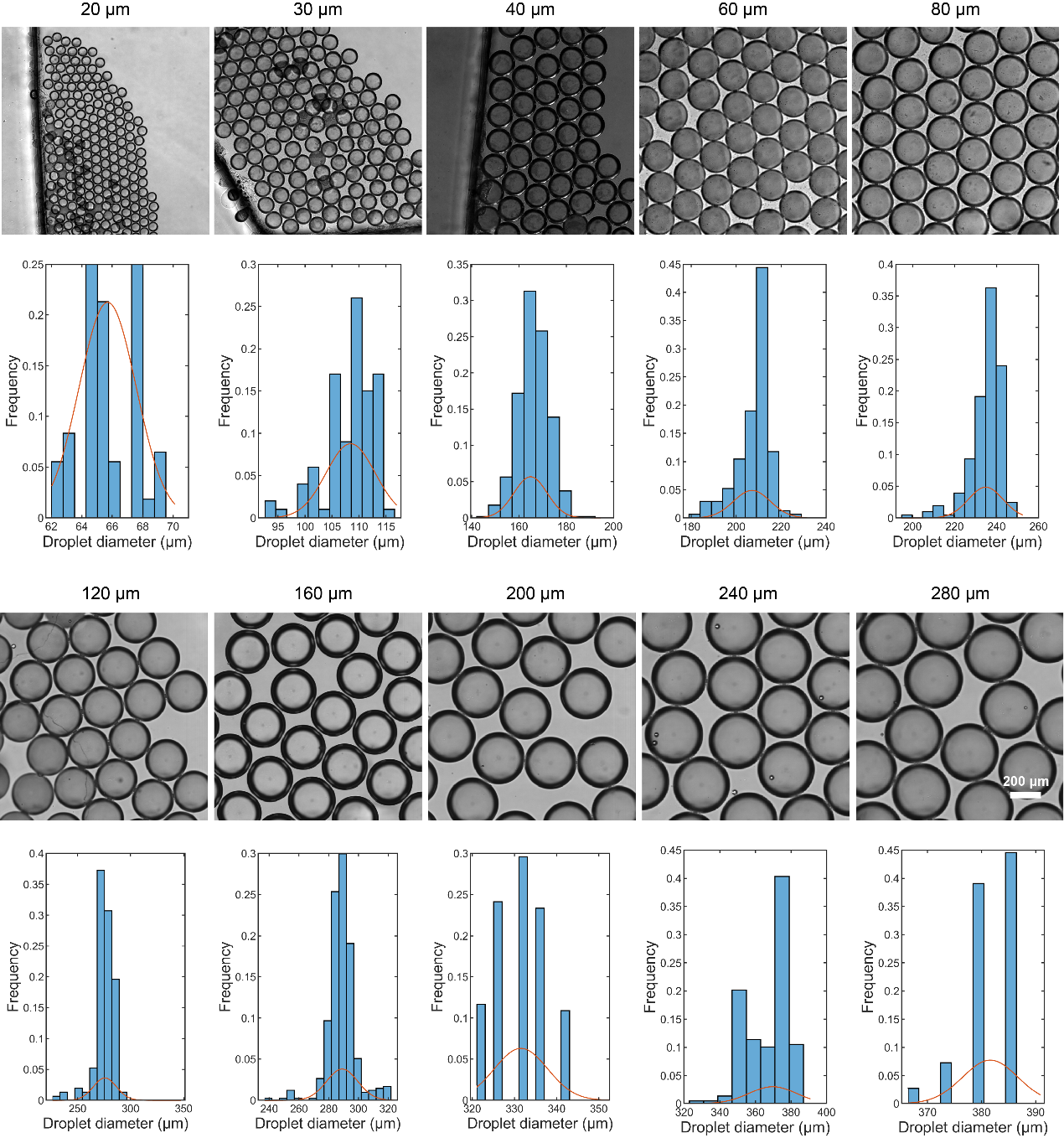


**Figure S23: Droplets generated with microchannels of different dimensions at 3000 rpm.**

## Supporting Tables:

**Table S1: Numerical values used for prediction of droplet generation.**

| **a (m s^-2^)** | $\boldsymbol{V}_{\boldsymbol{in}}$ **(m s^-1^)** | | | $\boldsymbol{R}_{\boldsymbol{2}}$ **(mm)** | **Rotational speed (rpm)** | **Volume of the aqueous phase (μL)** |
| --- | --- | --- | --- | --- | --- | --- |
|  | **Microchannel dimension (μm)** | | |  |  |  |
|  | **20** | **30** | **40** |  |  |  |
| **1052.8** | 0.015 | 0.037 | 0.068 | 7.5 | 2000 | 84 |
|  | 0.025 | 0.058 | 0.105 | 12.0 | 1835 | 302 |
|  | 0.034 | 0.079 | 0.142 | 16.9 | 1696 | 538 |
|  | 0.043 | 0.099 | 0.179 | 22.1 | 1577 | 788 |
|  | 0.052 | 0.120 | 0.216 | 27.5 | 1477 | 1047 |
| **2368.7** | 0.039 | 0.089 | 0.161 | 7.5 | 3000 | 84 |
|  | 0.059 | 0.136 | 0.244 | 12.0 | 2753 | 302 |
|  | 0.080 | 0.183 | 0.328 | 16.9 | 2543 | 538 |
|  | 0.101 | 0.230 | 0.411 | 22.1 | 2366 | 788 |
|  | 0.122 | 0.277 | 0.495 | 27.5 | 2216 | 1047 |
| **4211.0** | 0.071 | 0.163 | 0.292 | 7.5 | 4000 | 84 |
|  | 0.108 | 0.246 | 0.440 | 12.0 | 3671 | 302 |
|  | 0.145 | 0.329 | 0.588 | 16.9 | 3391 | 538 |
|  | 0.182 | 0.413 | 0.736 | 22.1 | 3155 | 788 |
|  | 0.219 | 0.496 | 0.884 | 27.5 | 2954 | 1047 |
| **6579.7** | 0.113 | 0.257 | 0.459 | 7.5 | 5000 | 84 |
|  | 0.171 | 0.387 | 0.691 | 12.0 | 4588 | 302 |
|  | 0.229 | 0.518 | 0.923 | 16.9 | 4239 | 538 |
|  | 0.287 | 0.648 | 1.154 | 22.1 | 3944 | 788 |
|  | 0.345 | 0.778 | 1.386 | 27.5 | 3693 | 1047 |

**Table S2: Parameters used in the CFD simulation.**

| **Name** | **Numerical value** | **Unit** |
| --- | --- | --- |
| **Geometry** |  |  |
| w&h | 20~40 | µm |
| l | 200 | µm |
| W&H | 400 | µm |
| L | 700 | µm |
| Ф1 | 20~40 | µm |
| Ф2 | 400 | µm |
| **Liquid condition** |  |  |
| Density of water | 998.2 | kg m^-3^ |
| Viscosity of water | 0.001003 | kg m^-1^ s^-1^) |
| Density of oil | 810.6 | kg m^-3^ |
| Viscosity of oil | 0.0094516 | kg m^-1^ s^-1^ |
| Surface tension coefficient | 0.00602 | N m^-1^ |
| **Other condition** |  |  |
| Contact angle of walls | 180 | deg |
| Inlet velocity | 0.015~1.39 | m s^-1^ |
| Outlet pressure | 0 | Pa |

**Table S3: Predictive results and valuation of the predictive model to experimental droplet dimension.**

| **Centrifugal acceleration**  **(m s^-2^)** | **Microchannel dimension**  **(w = h, μm)** | | **Flow rate (m s^-1^)** | **Experimental result (μm)** | **Predictive result (μm)** | **Error rate (%)** |
| --- | --- | --- | --- | --- | --- | --- |
| 1052.8 | | 20 | 0.015 | 94.5 | 96.0 | 1.64 |
| 1052.8 | | 20 | 0.034 | 174.9 | 159.3 | 8.91 |
| 1052.8 | | 30 | 0.058 | 261.1 | 244.6 | 6.32 |
| 1052.8 | | 40 | 0.105 | 328.1 | 325.1 | 0.92 |
| 1052.8 | | 40 | 0.216 | 400.3 | 388.7 | 2.89 |
| 2368.7 | | 20 | 0.039 | 115.4 | 139.3 | 20.70 |
| 2368.7 | | 20 | 0.080 | 189.7 | 192.1 | 1.29 |
| 2368.7 | | 30 | 0.136 | 257.6 | 265.0 | 2.88 |
| 2368.7 | | 40 | 0.244 | 311.2 | 332.7 | 6.90 |
| 2368.7 | | 40 | 0.495 | 392.8 | 385.5 | 1.85 |
| 4211.0 | | 20 | 0.071 | 148.6 | 137.3 | 7.61 |
| 4211.0 | | 20 | 0.145 | 185.5 | 179.3 | 3.33 |
| 4211.0 | | 30 | 0.246 | 261.2 | 238.6 | 8.66 |
| 4211.0 | | 40 | 0.440 | 296.8 | 293.8 | 1.00 |
| 4211.0 | | 40 | 0.884 | 315 | 336.3 | 6.76 |
| 6579.7 | | 20 | 0.113 | 152.4 | 156.1 | 2.43 |
| 6579.7 | | 20 | 0.229 | 194.2 | 191.7 | 1.27 |
| 6579.7 | | 30 | 0.387 | 233.1 | 241.8 | 3.73 |
| 6579.7 | | 40 | 0.691 | 293.2 | 288.2 | 1.69 |

**Table S4: The target sequence and primers used for LAMP.**

|  | **Sequence** |
| --- | --- |
| ***malB* gene** | GGATTTAAGCCATCTCCTGATGACGCATAGTCAGCCCATCATGAATGTTGCTGTCGATGACAGGTTGTTACAAAGGGAGAAGGGCATGGCGAGCGTACAGCTGCAAAATGTAACGAAAGCCTGGGGCGAGGTCGTGGTATCGAAAGATATCAATCTCGATATCCATGAAGGTGAATTCGTGGTGTTTGTCGGACCGTCTGGCTGCGGTAAATCGACTTTACTGCGCATGATTGCCGGGCTTGAGACGATCACCAGCGGCGACCTGTTCATCGGTGAGAAACGGATGAATGACACTCCGCCAGCAGAACGTGGCGTTGGTATGGTGTTTCAGTCCTACGCGCTCTATCCCCACCTGTCAGTAGCAGAAAACATGTCATTTGGCCTGAAACTGGCAGGCGCAAAAAAAGAGGTGATTAACCAACGCGTCAACCAGGTGGCGGAAGTGCTACAACTGGCGCATTTGCTGGATCGCAAACCGAAAGCGCTCTCCGGTGGTC |
| **F3** | GGCGTTGGTATGGTGTTTCA |
| **B3** | CAATCGCCACACGCTGAC |
| **FIP** | CCAGCCAGTTTCAGGCCAAATGCTTACGCGCTCTATCCCCA |
| **BIP** | GCGTTAACCAGGTGGCGGAAGCTTTCGGTTTGCGATCCA |
| **LF** | ACATGTTTTCTGCTACTGACAGG |
| **LB** | GTGCTACAACTGGCGCATTT |

**Table S5: 10× LAMP primers mixture used in ddLAMP.**

| **Primer** | **Volume（μL）** |
| --- | --- |
| F3 | 1 |
| B3 | 1 |
| FIP | 8 |
| BIP | 8 |
| LF | 2 |
| LB | 2 |
| H_2_O | 28 |
| Total | 50 |

**Table S6: Reagent mixture used for ddLAMP.**

| **Reagents** | **Working concentration** | **Storage concentration** | **Volume（μL）** |
| --- | --- | --- | --- |
| Buffer | 1× | 10× | 2 |
| Betaine | 1 M | 5 M | 4 |
| MgSO4 | 6 mM | 100 mM | 1.2 |
| dNTPs | 1.4 mM | 10 mM | 2.8 |
| Primer mixture | 1× | 10× | 2 |
| BSA | 1 mg mL^-1^ | 20 mg mL^-1^ | 1 |
| Evagreen | 1× | 20× | 1 |
| H_2_O |  |  | 4 |
| Bst 3.0 | 0.4 U μL ^-1^ | 8 U μL ^-1^ | 1 |
| Template |  |  | 1 |
| Total |  |  | 20 |

## Supporting Text:

**Text S1. Three strategies for droplet tiling in the CASE.**

To enhance the applicability of the CASE for various droplet microfluidic applications, it is desirable to tile the stacked droplets for better observation and analysis. Unlike droplets generated in heavy oil by centrifugal force, which tend to spread and fulfill the oil surface in a single-layer format, those generated in light oil prefer to stack at the bottom of the droplet reservoir layer by layer. However, the surfactant used in heavy oil is over a thousand times more expensive than that used in light oil. To save cost and address the stacking issue, researches have proposed several solutions such as providing extra oil storage structures beforehand in droplet reserviors^[1]^, inverting and restricting the sinking space to single layer droplet diamensions^[2]^, employing 3D scanning and image reconstruction^[3,4]^, or directly switching to an outside counting plate^[5,6]^. However, most of these solutions involve intricate microfluidic structures, elaborate instrumentation, or disruptive manual operations, which hinder automation and necessitate excessive costs and trained personnel. Therefore, to integrate thorough observation of droplets in the CASE, we proposed three droplet tiling strategies: inverting, inward and outward strategy, each with individually designed microfluidic chips.

The driving force behind droplet tiling is crucial for ensuring that stacked droplets after generation disperse evenly into a broader area. Since there's no inherent centripetal force available, we utilized gravity and centrifugal force to achieve this (Figure S15a, Supporting Information). In the inverting strategy, gravity plays a significant role. By sloping the chip, the stacked droplets in the distal and higher positions naturally sank downwards into the broader space below. The component force of gravity parallel to the bottom surface drove droplets towards the proximal position, while the vertical component force pushed upper droplets downward. Additionally, repulsive forces between droplets, caused by surfactants, created extra space for upper droplets during transit. These leaded to the formation of single-layer droplets within 5 minutes (Figure S15b, S16, Supporting Information). However, due to the fixed direction of gravity, only one CASE can perform droplet tiling at a time in a microfluidic disk with multiple CASEs, limiting its application for high-throughput experiments.

To further expedite the droplet tiling process and enhance throughput, we introduced the inward strategy, which involves isolating a CASE within a cartridge chip. This cartridge chip was securely fixed and driven within the centrifugal field using a positioner equipped with multiple chip stationary areas (Figure S15c, S17, Supporting Information). In this setup, cartridge chips can be loaded into chip stationary areas of identical dimensions via a chip loading area. With the aqueous phase positioned proximally and the oil phase distally, droplets can be efficiently generated at high rotational speeds (Figure S15d, Supporting Information). Subsequently, the cartridge chips can be removed, rotated by 180°, and then reloaded, the distal droplets were thus exchanged to proximal position with respect to the rotational center. At a low rotational speed, the droplets can be tiled two minutes faster than that utilizing the inverting strategy because of the higher centrifugal force and gravity used.

The outward strategy involves imposing a restriction in a proximal position during droplet generation and then removing it to release the droplets into the distal end. To implement this strategy, we designed a concentric inclined plane as the restriction, connecting the nozzles at the same horizontal level to the droplet reservoir in the upper horizontal level (Figure S15e-g, Supporting Information). In this setup, the droplet reservoir was initially filled with oil phase by driving the oil deposited in an oil reservoir beforehand (Figure S3b, Supporting Information). During emulsification, the droplets were obstructed by the concentric inclined plane. After emulsification, the chip was turned over, allowing the droplets to sink to the droplet reservoir along the inclined plane. Within a few minutes, all the droplets stacked in the droplet reservoir, enabling the application of centrifugal force for droplet tiling, similar to the inward strategy. The outward strategy enables high-throughput droplet tiling with limited manual intervention, making it a valuable approach for various droplet microfluidic applications. In summary, these three strategies provide efficient methods for droplet tiling generated by a CASE. Thereby, an all-in-one detection from droplet pre-design, generation, manipulation and observation can be performed in the CASE. The fundamental methodologies underlying these strategies offer valuable guidance for other centrifugal droplet platforms utilizing light oil.

**Supporting References**

[1] H. Peng, M. Zhu, Z. Gao, C. Liao, C. Jia, H. Wang, H. Zhou, J. Zhao, *Biomed. Microdevices* **2020**, *22*, 18.

[2] M. Jiang, P. Liao, Y. Sun, X. Shao, Z. Chen, P. Fei, J. Wang, Y. Huang, *Lab Chip* **2021**, *21*, 2265.

[3] P. Liao, M. Jiang, Z. Chen, F. Zhang, Y. Sun, J. Nie, M. Du, J. Wang, P. Fei, Y. Huang, *Proc. Natl. Acad. Sci. U.S.A.* **2020**, *117*, 25628.

[4] E. Y. Shum, J. H. Lai, S. Li, H. G. Lee, J. Soliman, V. K. Raol, C. K. Lee, S. P. A. Fodor, H. C. Fan, *Anal. Chem.* **2022**, 94, 17868.

[5] S. Ye, C. Li, X. Zheng, W. Huang, Y. Tao, Y. Yu, L. Yang, Y. Lan, L. Ma, S. Bian, W. Du, *Anal. Chem.* **2022**, *94*, 2918.

[6] Z. He, J. Wang, B. J. Fike, X. Li, C. Li, B. L. Mendis, P. Li, *Biosens. Bioelectron.* **2021**, *191*, 113458.
